# Disease modeling of core pre-mRNA splicing factor haploinsufficiency

**DOI:** 10.1093/hmg/ddz169

**Published:** 2019-07-13

**Authors:** Katherine A Wood, Charlie F Rowlands, Wasay Mohiuddin Shaikh Qureshi, Huw B Thomas, Weronika A Buczek, Tracy A Briggs, Simon J Hubbard, Kathryn E Hentges, William G Newman, Raymond T O’Keefe

**Affiliations:** 1 Division of Evolution and Genomic Sciences, School of Biological Sciences, Faculty of Biology, Medicine and Health, The University of Manchester; 2 Center for Genomic Medicine, Division of Evolution and Genomic Sciences, School of Biological Sciences, Faculty of Biology, Medicine and Health, St. Mary’s Hospital, The University of Manchester, Manchester Academic Health Science Centre Manchester, M13 9PT, UK

## Abstract

The craniofacial disorder mandibulofacial dysostosis Guion-Almeida type is caused by haploinsufficiency of the U5 snRNP gene *EFTUD2/SNU114*. However, it is unclear how reduced expression of this core pre-mRNA splicing factor leads to craniofacial defects. Here we use a CRISPR-Cas9 nickase strategy to generate a human *EFTUD2*-knockdown cell line and show that reduced expression of *EFTUD2* leads to diminished proliferative ability of these cells, increased sensitivity to endoplasmic reticulum (ER) stress and the mis-expression of several genes involved in the ER stress response. RNA-Seq analysis of the *EFTUD2*-knockdown cell line revealed transcriptome-wide changes in gene expression, with an enrichment for genes associated with processes involved in craniofacial development. Additionally, our RNA-Seq data identified widespread mis-splicing in *EFTUD2*-knockdown cells. Analysis of the functional and physical characteristics of mis-spliced pre-mRNAs highlighted conserved properties, including length and splice site strengths, of retained introns and skipped exons in our disease model. We also identified enriched processes associated with the affected genes, including cell death, cell and organ morphology and embryonic development. Together, these data support a model in which *EFTUD2* haploinsufficiency leads to the mis-splicing of a distinct subset of pre-mRNAs with a widespread effect on gene expression, including altering the expression of ER stress response genes and genes involved in the development of the craniofacial region. The increased burden of unfolded proteins in the ER resulting from mis-splicing would exceed the capacity of the defective ER stress response, inducing apoptosis in cranial neural crest cells that would result in craniofacial abnormalities during development.

## INTRODUCTION

Precursor messenger RNA (pre-mRNA) splicing—the removal of introns followed by the joining of exons to form a continuous open reading frame (ORF) within a mature mRNA transcript—is a fundamental prerequisite for the translation of mRNAs into proteins in eukaryotic cells. Splicing is carried out by a large dynamic ribonucleoprotein complex known as the spliceosome, which consists of five small nuclear RNAs (snRNAs) complexed with proteins to form small nuclear ribonucleoprotein complexes (snRNPs) and almost 200 auxiliary proteins (1–3). The majority of human introns (approximately 95.5%) are spliced by the major spliceosome, which contains the U1, U2, U4, U5 and U6 snRNPs. The minor, or U12-dependent, spliceosome is formed of the U11, U12, U4atac, U5 and U6atac sRNPs and is responsible for the splicing of approximately 800 introns (4). In both cases, splicing occurs in multiple steps, in which the spliceosomal machinery is recruited to *cis-*acting sequences within the pre-mRNA itself, namely the 5′ and 3′ splice sites (5′SS and 3′SS, respectively) at either end of the intron, and the branch point sequence (BPS), containing a highly conserved adenosine, a short distance upstream of the 3′SS (5). Splicing occurs in two sequential transesterification reactions, resulting in the removal of the intron as a branched lariat structure and the joining of the two exons together (1,2).

Variants in the spliceosomal machinery have been identified as causing several human cancers and other genetic disorders (6–8). Although pre-mRNA splicing is a ubiquitous and fundamental process occurring in all human cells, these disorders tend to have restricted and specific phenotypes. For example, variants in at least eight core spliceosomal genes are associated with autosomal dominant retinitis pigmentosa (adRP), one of the leading causes of hereditary blindness, while variants in *SNRPE* are linked to isolated hypotrichosis (9–12). Of interest here are variants in five core spliceosome factors that are associated with a group of human disorders in which patients display abnormal craniofacial development as the primary phenotype, with developmental delay and/or skeletal defects commonly observed as additional phenotypes (13). These disorders suggest that craniofacial development is particularly sensitive to abnormalities in pre-mRNA splicing, although the underlying mechanisms behind this sensitivity remain unclear. Currently, five such disorders have been identified—mandibulofacial dysostosis Guion-Almeida type (MFDGA), Burn–McKeown syndrome (BMKS), Nager syndrome (NS), Richieri–Costa Pereira syndrome (RCPS) and cerebrocostomandibular syndrome (CCMS) (13). Of particular interest, both MFDGA and BMKS are caused by variants in U5 snRNP-specific genes, suggesting an involvement of the U5 snRNP in craniofacial development and a potential link between the causative genes in these disorders (14,15). The contrast between the tissue-specific disease phenotypes arising from variants in different core spliceosome genes, even between factors that are found in the same splicing complex, is remarkable. However, variants in the splicing factor CWC27 have now been identified as presenting with RP, craniofacial defects and developmental delay, suggesting that the overlap of distinct disease phenotypes is possible (16).

MFDGA patients characteristically display malar and mandibular hypoplasia, microcephaly, external ear malformations and intellectual disability. Other features, such as hearing loss, cleft palate, choanal atresia, oesophageal atresia, congenital heart defects and radial ray defects are also (less commonly) observed (17). Several studies have reported heterozygous loss-of-function variants in the U5 snRNP component *EFTUD2* as the underlying pathogenic cause of MFDGA. These studies have revealed a variety of different *EFTUD2* variants in MFDGA patients, including missense variants, frameshifts, nonsense variants and splice site variants, all of which are predicted to inactivate one allele and thus reduce *EFTUD2* expression leading to haploinsufficiency as the underlying mechanism of disease (15,18–20).

*EFTUD2* encodes a GTPase that is essential during multiple steps of the spliceosomal cycle, and is highly conserved across eukaryotes from yeast to humans (21). Snu114p, the *Saccharomyces cerevisiae* ortholog of human EFTUD2, has been shown to play critical roles in spliceosome remodeling and dynamics during pre-mRNA splicing (22). Snu114p interacts genetically and physically with the RNA helicase Brr2p and Prp8p (23–26). Similarly, in humans, physical interactions have been demonstrated between EFTUD2, SNRNP200 (Brr2p ortholog) and PRPF8 (Prp8p ortholog), which have been confirmed by recent cryo-EM structures of the human U4/U6.U5 tri-snRNP (27,28). Prior to the first catalytic step of splicing, Snu114p is involved in the dissociation of the U4 and U6 snRNAs by regulating the ATPase activity of Brr2p, while after the splicing reactions are complete Snu114p is believed to regulate the dissociation of spliceosomal subunits (27,29–31). Interestingly, variants in both *SNRNP200* and *PRPF8* are associated with adRP and not craniofacial defects, highlighting the phenotypic discrepancies arising from variants in different but interacting components of the same core spliceosomal complex.

Work from our laboratory has investigated the effects of reduced *SNU114* expression in *S. cerevisiae* (Thomas *et al.*, *in preparation*). Additionally, several groups have generated zebrafish models in which the *eftud2* gene is disrupted (32,33). These models have demonstrated an important involvement of *eftud2* in neural progenitor development and possible links between *EFTUD2* variants, endoplasmic reticulum (ER) stress, an over-burdened non-sense-mediated decay (NMD) pathway and aberrantly activated p53 in neural progenitors. However, until now, there have been no reports directly investigating the effects of reduced *EFTUD2* expression in human cells.

Here, we report the generation and characterization of a novel human HEK293 cell line, the 3.C9 cell line, with a heterozygous loss-of-function mutation in *EFTUD2*, which displayed proliferation defects and an increased sensitivity to ER stress. Furthermore, whole transcriptome RNA-Seq revealed transcriptome-wide defects in gene expression and pre-mRNA splicing in this mutant cell line, while highlighting key networks and pathways particularly affected by *EFTUD2* haploinsufficiency and features of introns and exons in which splicing was disrupted when *EFTUD2* expression was reduced. Taken together, this first report of the effects of reduced *EFTUD2* expression in a human cell line enhances our understanding of the importance of *EFTUD2* in human cells and begins to unravel how *EFTUD2* haploinsufficiency leads to craniofacial defects in humans.

## Results

### CRISPR-Cas9 nickase-mediated *EFTUD2* disruption in human cells

**Figure 1:**
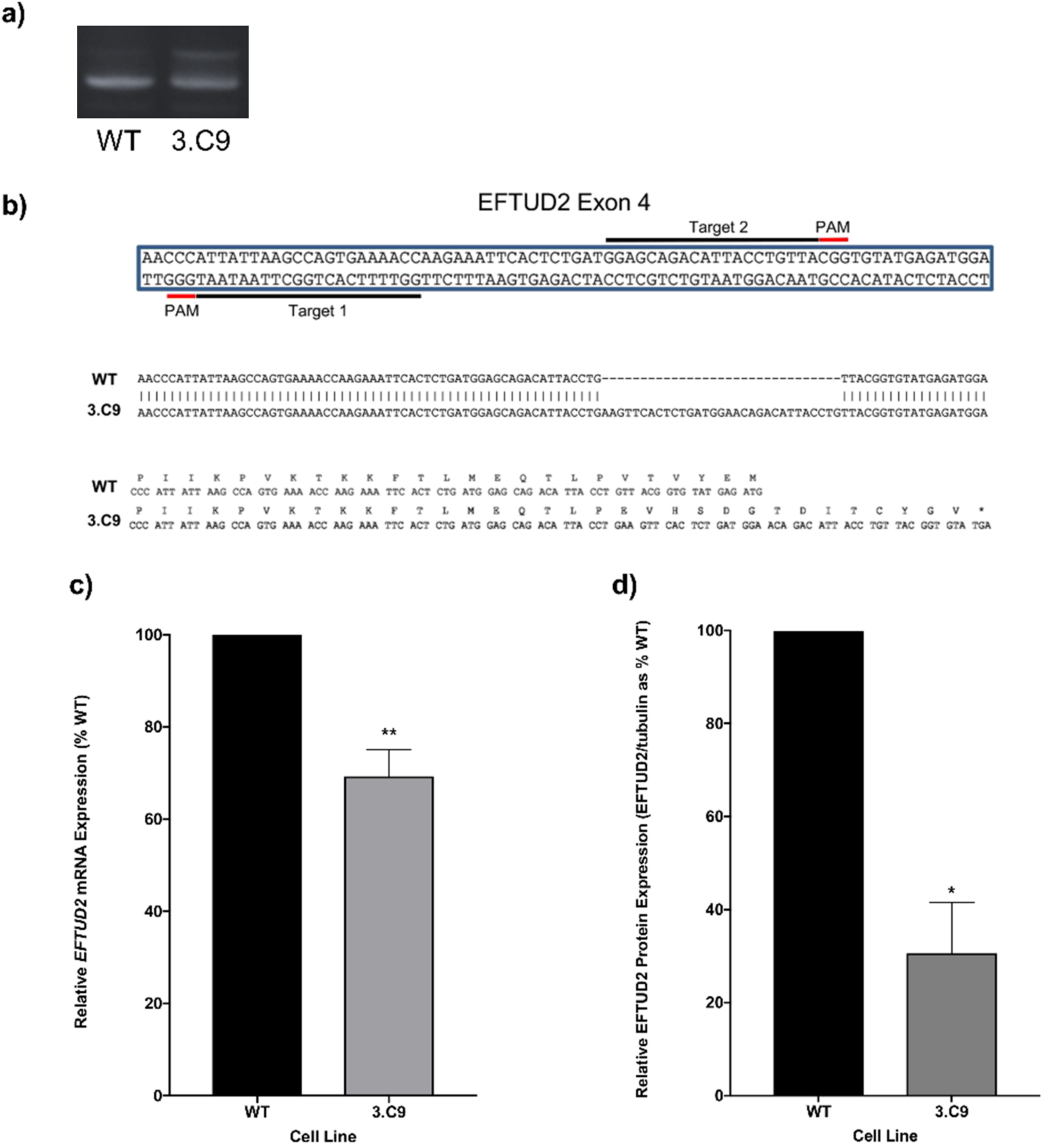
Generation of an *EFTUD2*-mutant HEK293 cell line using the CRISPR-Cas9 nickase strategy. (**a**) Amplification of genomic DNA from WT HEK293 cells and the 3.C9 *EFTUD2*-mutant cell line by PCR using primers targeting *EFTUD2* exon 4. Genomic DNA from the 3.C9 cell line gives two distinct bands, corresponding to the WT allele (lower band) and a mutant allele containing an insertion (upper band) compared to the single, lower band for the WT cells. (**b**) Target sites for the guide RNAs (gRNAs) used to target the Cas9 nickase to *EFTUD2* exon 4 using the CRISPR strategy and the corresponding DNA sequence and protein sequence alignments for *EFTUD2* in WT and 3.C9 cells. The 3.C9 cells contain a 31 bp insertion in *EFTUD2* exon 4 on one allele, generating a frame shift that results in a premature stop codon. (**c**) Relative mRNA expression levels for the *EFTUD2* in 3.C9 cells compared to WT cells, determined using qPCR of cDNA from each cell line. Graphs were obtained using the ΔΔC_T_ method with *ACTB* as the endogenous reference gene. *n* = 5. ^**^p-value < 0.01. (**d**) Relative protein levels for EFTUD2 in 3.C9 cells compared to WT cells, determined using western blotting of total cell extracts. Signals were quantified using LI-COR Image Studio software, normalizing the EFTUD2 signal to α-tubulin, and values expressed as percentages of WT controls. Graphs show standard error of the mean of four experiments. ^*^p-value < 0.05.

To investigate the effects of reduced *EFTUD2* expression levels in human cells, a CRISPR-Cas9 nickase genome engineering strategy was employed to generate stable human cell lines containing mutations in *EFTUD2* (34). The Cas9 nickase (Cas9n) system was selected for genome disruption over the canonical Cas9 system as Cas9n reduces off-target effects by up to 1500-fold by producing only single-strand breaks and requiring two nearby independent binding events ensuring high specificity genome editing (35–37). HEK293 cells were selected for genome editing for their ease of transfection and efficiency of CRISPR-Cas9 genome editing, making them an ideal system for initial analysis before moving into more complex systems such as patient-derived induced pluripotent stem cells (iPSCs) (38). CRISPR-Cas9-mediated genome engineering in HEK293 cells has been used for initial studies of other disorders such as Huntington’s disease and lung adenocarcinoma (39–41). Guide RNA pairs were designed to target *EFTUD2* exon 4 encoding the N-terminus of EFTUD2, upstream of the G domain (chr17:44885269 using the hg38/GRCh38 reference genome). After transfecting SpCas9 nickase-chimeric gRNA expression vectors into HEK293 cells, cells were plated at approximately one cell per well in a 96-well plate format to obtain clones derived from a single transfected cell. Potential clones were identified by PCR screening of genomic DNA. One clone (3.C9) was selected for further analysis (Fig. 1a). Sequencing of PCR products derived from the region of exon 4 targeted in the 3.C9 cell line gave rise to not only the wildtype (WT) *EFTUD2* sequence but also a distinct sequence in which 31 base-pairs (bp) were inserted into *EFTUD2* exon 4. This 31 bp insertion generates a frameshift in the ORF, introducing a premature termination codon (PTC) soon after the site of insertion (Fig. 1b). This PTC is predicted to activate NMD of the pre-mRNA producing a null allele, rather than the production of a truncated protein, as the PTC is greater than 50 nucleotides (nt) from the last exon junction complex (EJC). We also do not detect any truncated forms of EFTUD2 in the 3.C9 cell line by western blotting. Therefore, the 3.C9 cell line has one WT allele and one allele in which *EFTUD2* is inactivated, recapitulating the observed frameshift mutations/null alleles observed in patients with MFDGA.

To determine whether the mutation introduced into *EFTUD2* in the 3.C9 cell line affected the expression of *EFTUD2*, quantitative PCR (qPCR) was performed. Expression of *EFTUD2* mRNA was significantly lower in 3.C9 cells, with expression reduced to 68% that of WT expression levels, as would be expected in the case of a heterozygous null allele (Fig. 1c). To investigate whether the reduced expression of *EFTUD2* mRNA in the 3.C9 cell line translated into changes in the levels of EFTUD2 protein, western blotting was employed to quantitate EFTUD2 protein levels. In the 3.C9 cells, the level of EFTUD2 protein was reduced to 31% that of WT cells (Fig. 1d). Furthermore, the western blots only revealed a single EFTUD2 band corresponding to full-length protein. This observation suggested that the 31 bp insertion in exon 4 of *EFTUD2* in 3.C9 cells that resulted in a PTC did indeed trigger NMD of the pre-mRNA as predicted, rather than escaping NMD to produce a truncated protein, making this 31 bp insertion allele a null allele equivalent to that observed in MFDGA patients.

**Figure 2:**
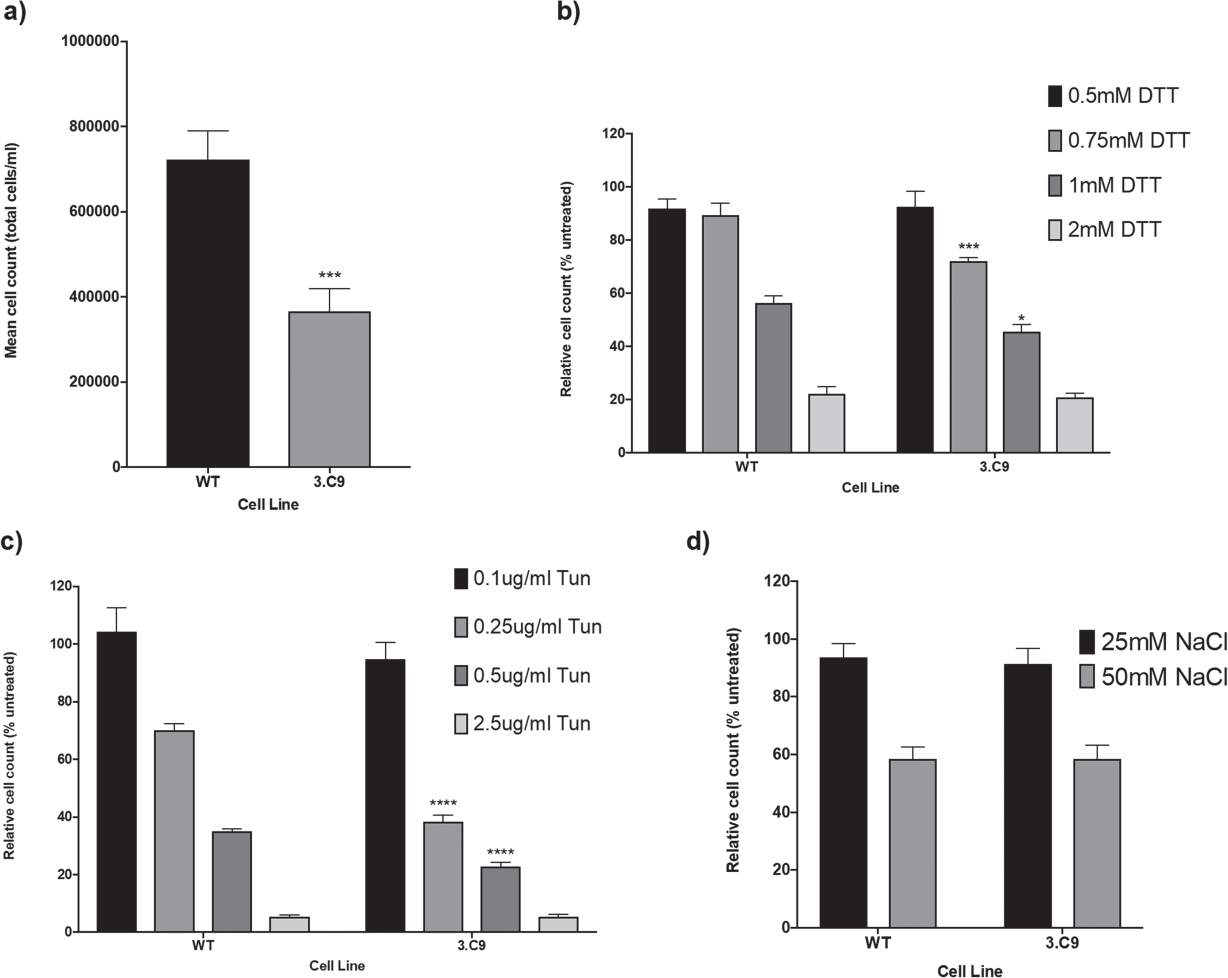
Cell line growth rates under normal and stress conditions. (**a**) Mean cell counts for WT HEK293 and 3.C9 cells after seeding at a density of 2.5 × 10^5^ cells per well and growth under normal culture conditions (37°C, 5% CO_2_) for 48 h. *n* = 8. ^***^p-value < 0.001. (**b**) Relative cell counts for WT and 3.C9 cells expressed as a percentage of the cell count for untreated cells following seeding at a density of 2.5 × 10^5^ cells per well and growth for 48 h in different concentrations of DTT. *n* = 8. ^*^p-value < 0.05, ^***^p-value < 0.001. (**c**) Relative cell counts for WT and 3.C9 cells expressed as a percentage of the cell count for untreated cells following seeding at a density of 2.5 × 10^5^ cells per well and growth for 48 h in different concentrations of tunicamycin (Tun). *n* = 8. ^****^p-value < 0.0001. (**d**) Relative cell counts for WT and 3.C9 cells expressed as a percentage of the cell count for untreated cells following seeding at a density of 2.5 × 10^5^ cells per well and growth for 48 h in different concentrations of sodium chloride (NaCl). *n* = 8.

To examine the proliferative fitness of the 3.C9 *EFTUD2*-knockdown cell line, cell counting assays were performed with cells seeded at identical densities and allowed to grow and proliferate for 48 h under normal cell culture conditions before counting with an automated cell counter. The 3.C9 cell line proliferated less than WT cells, with a relative cell count only 52% that of WT cells after 48 h of growth (Fig. 2a). Analysis of histone H3 serine 10 phosphorylation to detect mitotic cells did not reveal a difference in the overall number of mitotic cells between WT and 3.C9 cells (Supplementary Material, Fig. S1). However, a significant increase in monopolar spindles with rosette-like chromosomes was observed in 3.C9 compared to WT cells indicative of a cell cycle defect (Supplementary Material, Fig. S1). Given the core nature of EFTUD2 in the spliceosome and the importance of pre-mRNA splicing in all cellular processes, it is perhaps unsurprising that when EFTUD2 expression is reduced, the ability of cells to proliferate and/or grow is compromised, although clearly the reduction in proliferation must be tolerable as MFDGA patients survive.

### Altered proliferation of *EFTUD2*-knockdown cells under conditions of stress

Concurrent work from our laboratory demonstrated that *SNU114* heterozygous *S. cerevisiae* models of MFDGA were sensitive to ER stress, displaying reduced growth fitness with three different ER stress-inducing agents [dithiothreitol (DTT), tunicamycin (Tun) and β-mercaptoethanol]. No differences in growth fitness under conditions of other cellular stresses, such as osmotic stress, oxidative stress, different pHs and different temperatures were observed with these models (Thomas *et al.*, *in preparation*). To determine whether the 3.C9 human cell line was also sensitive to ER stress, proliferation assays were performed with cells treated with ER stress-inducing agents at various concentrations (Fig. 2b and c).

We studied the fitness of 3.C9 cells in four different concentrations of DTT (Fig. 2b). Growth of 3.C9 and WT cells was only slightly affected by 0.5 mm DTT, with relative cell counts of 92% and 93% that of untreated cells for WT and 3.C9 cells, respectively. On the other hand, 2 mm DTT treatment appeared to be a relatively lethal concentration for both 3.C9 and WT cells, with the relative cell counts only 22% and 21% that of untreated cells for WT and 3.C9 cells, respectively. These findings suggested that at low and high DTT concentrations, there was little difference in growth fitness between WT and 3.C9 cell lines. However, at the intermediate 0.75 mm DTT concentration, there were significant (*P* < 0.001) differences between WT cells and 3.C9 cells, with the WT cell count at 89% that of untreated cells and the 3.C9 cell count at 72% that of untreated cells. This significantly increased sensitivity of the 3.C9 cells to DTT was also observed at 1 mm DTT, with relative cell counts of 56% that of untreated cells for WT but only 45% that of untreated cells for the 3.C9 line (*P* < 0.05). Therefore, at intermediate but sub-lethal concentrations of DTT, the 3.C9 cell line displayed reduced fitness.

To confirm that the increased sensitivity of the 3.C9 cell line in DTT was a result of ER stress and not a drug-specific effect, the growth assay was repeated using a second ER stress-inducing agent, Tun (Fig. 2c). At 0.1 μg/ml Tun, there were no significant differences in the relative cell counts for WT and 3.C9 cells. However, at 0.25 μg/ml Tun, there was a significant (*P* < 0.0001) difference between the two cell lines, with WT cells having a relative cell count of 70% while for 3.C9 cells the relative cell count was just 38%. Similarly, at 0.5 μg/ml Tun there was a significant (*P* < 0.0001) difference in the response of the two cell types; WT cells had a relative cell count of 35% while for 3.C9 the relative cell count was 23%. At 2.5 μg/ml, a lethal concentration of the drug, the relative cell count was reduced to 5% for both cell types. Therefore, over a range of concentrations of Tun, the proliferation of 3.C9 cells was more affected than that of WT cells, most prominently at 0.25 μg/ml Tun. Together, these cell-counting results support evidence for an increased sensitivity of 3.C9 cells to ER stress.

To confirm that 3.C9 cells were specifically sensitive to ER stress, cell counting was repeated with two concentrations of NaCl to induce osmotic stress (Fig. 2d). At both NaCl concentrations, there were no significant differences between the relative cell counts for WT and 3.C9 cells. At 25 mm NaCl, the relative cell count for WT cells was 94%, compared to 92% for 3.C9 cells. At 50 mm NaCl, the relative WT cell count was reduced to 58% while for the 3.C9 cells the relative cell count was 59%. Thus, in contrast to the notable differences between the relative cell counts for the two cell lines in both ER stresses DTT and Tun, NaCl did not have a differential effect on 3.C9 proliferation compared to WT cells.

### ER stress response gene expression in *EFTUD2*-knockdown cells

**Figure 3:**
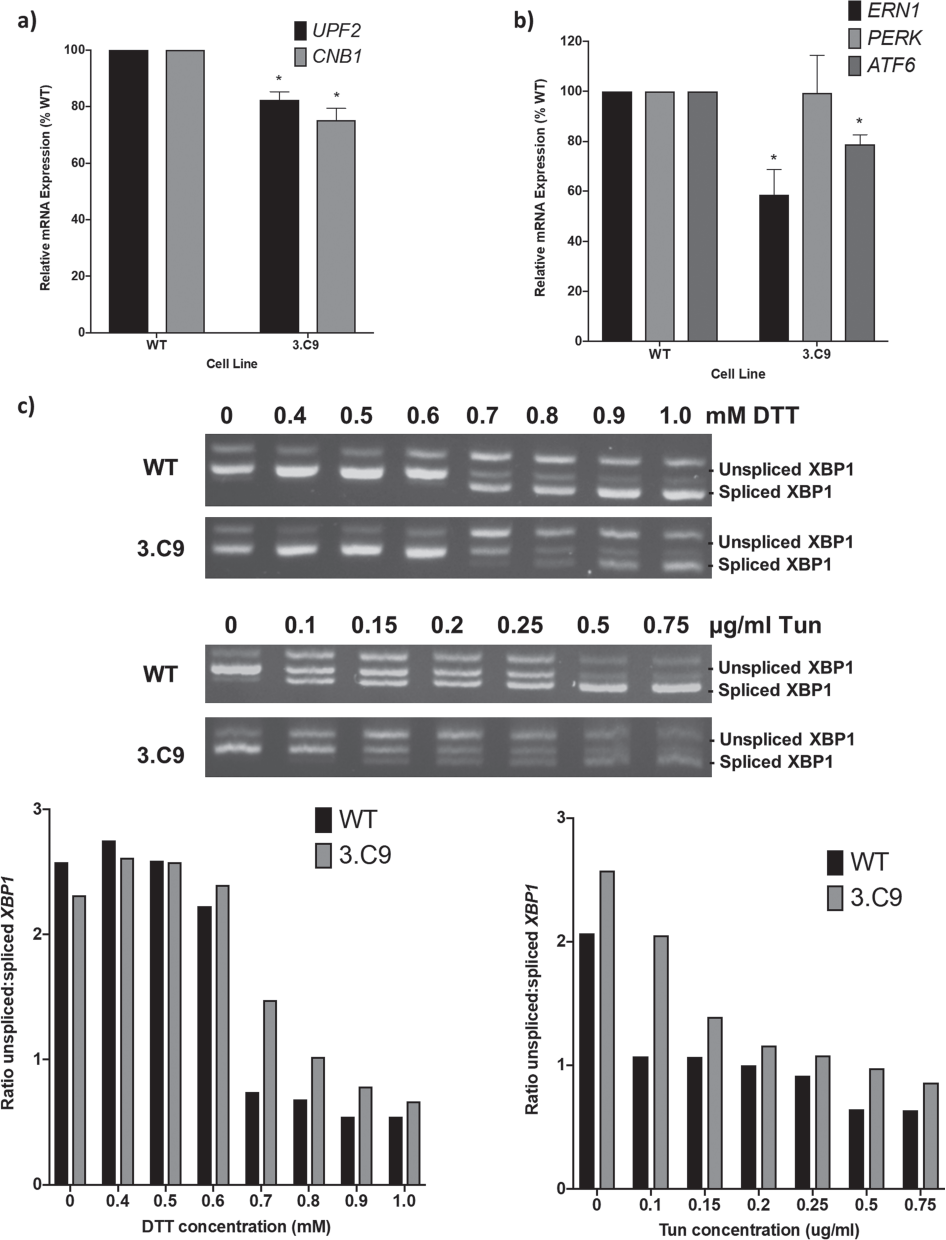
Defects in the endoplasmic reticulum stress response in *EFTUD2*-mutant cells. (**a**) Relative mRNA expression levels for the endoplasmic stress (ER) response genes *CNB1* and *UPF2* in 3.C9 cells compared to WT HEK293 cells, determined using qPCR with cDNA made from the cell lines. Graphs were obtained using the ΔΔC_T_ method with *ACTB* as the endogenous reference gene. *n* = 4. ^*^p-value < 0.05. (**b**) Relative mRNA expression levels for the ER stress response genes *ERN1, PERK* and *ATF6* in 3.C9 cells compared to WT cells, determined using qPCR with cDNA made from the cell lines. Graphs were obtained using the ΔΔC_T_ method with *ACTB* as the endogenous reference gene. *n* = 5. ^*^p-value < 0.05. (**c**) *XBP1* splicing in WT and 3.C9 cells following 5 h DTT (above) and Tun (below) treatment at increasing concentrations. cDNA was made from cells lines following 5 h growth at 37°C in 5% CO_2_ with and without indicated agents and used as a template in RT-PCR reactions with primers that amplify both the spliced and unspliced forms of *XBP1*. Relative intensities of spliced and unspliced XBP1 were quantified using ImageJ software.

To determine whether cellular stress response genes were influenced by *EFTUD2* knockdown in the human 3.C9 disease model cell line, qPCR analysis was employed to investigate the mRNA expression levels for *CNB1* and *UPF2*, genes involved in the ER stress and unfolded protein response (UPR) (42). Both *CNB1* and *UPF2* mRNA expression was significantly reduced in the 3.C9 cells compared to the WT cells (Fig. 3a). In 3.C9 cells, mean *UPF2* expression was reduced to 82% while *CNB1* expression was 75% that of WT levels. This reduced mRNA expression of *UPF2* and *CNB1* could explain the increased sensitivity of 3.C9 cells to ER stressing agents.

In mammals, there are three signaling branches of the ER stress and UPR, namely, the IRE1α pathway, the PERK pathway and the ATF6 pathway (43–45). Together, these UPR programmes activate hundreds of genes to overcome ER stress, but when the system fails to restore homeostasis, it can trigger apoptosis (46). To investigate expression of key UPR genes in the *EFTUD2*-knockdown cell line, the mRNA expression of *ERN1* (encoding IRE1α), *PERK* and *ATF6* was probed by qPCR. Expression of both *ERN1* and *ATF6* was significantly reduced in 3.C9 cells compared to WT cells, while *PERK* expression was not significantly changed (Fig. 3b). Thus, the expression of key players in two of the three mammalian UPR branches were altered when *EFTUD2* levels are reduced in the 3.C9 cell line, which, along with the reduced expression of *CNB1* and *UPF2*, could account for the compromised response to induced ER stress in these cells.

The IRE1α branch of the UPR has been well documented in mammalian cells. IRE1α resides in the ER membrane and is activated by oligomerization and autophosphorylation in response to ER stress (47). Activated IRE1α then catalyses the unconventional, spliceosome-independent, intron removal from the *XBP1* pre-mRNA, allowing production of active XBP1, which induces expression of other genes involved in the ER stress response (48). To determine whether *XBP1* splicing was compromised by reduced *ERN1* (IRE1α) expression in 3.C9 cells, reverse transcription (RT)-PCR was used to detect both spliced and unspliced *XBP1* isoforms in 3.C9 and WT cells cultured in different concentrations of ER stress-inducing agents (Fig. 3c). Under normal culture conditions without DTT or Tun, there were no differences between WT cells and the 3.C9 *EFTUD2* mutant cell line (Fig. 3c). At DTT concentrations of 0.6 mm and lower, only a single PCR product, corresponding to the unspliced form of *XBP1*, was observed in both WT and 3.C9 cells, suggesting that at these low concentrations of ER stressing agent, the IRE1α/XBP1 branch of the UPR is not significantly activated. However, at 0.7 mm and 0.8 mm DTT, spliced *XBP1* was observed for WT cells, while for 3.C9 cells there was only a very faint band corresponding to the spliced product, so the ratio of unspliced *XBP1* to spliced *XBP1* is markedly lower for WT cells than for 3.C9 cells (Fig. 3c). At 0.9 mm and 1 mm DTT, the majority of *XBP1* appeared in its spliced form in both cell types, although the ratio of unspliced:spliced *XBP1* remained higher in the 3.C9 cells. These results suggest that the 3.C9 *EFTUD2* mutant cell line displays delayed splicing of *XBP1*, requiring higher concentrations of DTT than WT cells to achieve the same level of *XBP1* splicing. This delayed *XBP1* splicing is likely to result from the significantly reduced expression levels of *ERN1* and could contribute to the increased ER stress sensitivity of the 3.C9 cells.

To confirm that the differences in *XBP1* splicing observed were a result of ER stress and not a specific effect of DTT, the assay was repeated by treating both cell types for 5 h with Tun at increasing concentrations (Fig. 3c). The WT cells had a lower ratio of unspliced *XBP1* to spliced *XBP1* than 3.C9 cells in the absence of Tun, but the addition of Tun at concentrations as low as 0.1 μg/ml induced a greater reduction in the unspliced *XBP1*:spliced *XBP1* ratio for the WT cells than 3.C9 cells (Fig. 3c). For the WT cells, at 0.1 μg/ml Tun the ratio of the two *XBP1* isoforms was approximately 1:1, while for 3.C9 cells, 0.25 μg/ml Tun was required to achieve a 1:1 ratio of the two products, and compared to WT cells the unspliced:spliced ratio of *XBP1* was higher at all Tun concentrations tested (Fig. 3c). Thus, as with DTT, *XBP1* splicing was delayed with Tun treatment in the *EFTUD*2 mutant cells, requiring higher concentrations of Tun to achieve the same level of *XBP1* splicing. To confirm that *XBP1* splicing is indeed specific to ER stress, the assay was repeated following 5 h of NaCl treatment at various concentrations to induce osmotic stress. For both WT and 3.C9 cells, a product corresponding to the unspliced isoform of *XBP1* was observed but there was no smaller product corresponding to the spliced form (Supplementary Material, Fig. S2).

**Figure 4:**
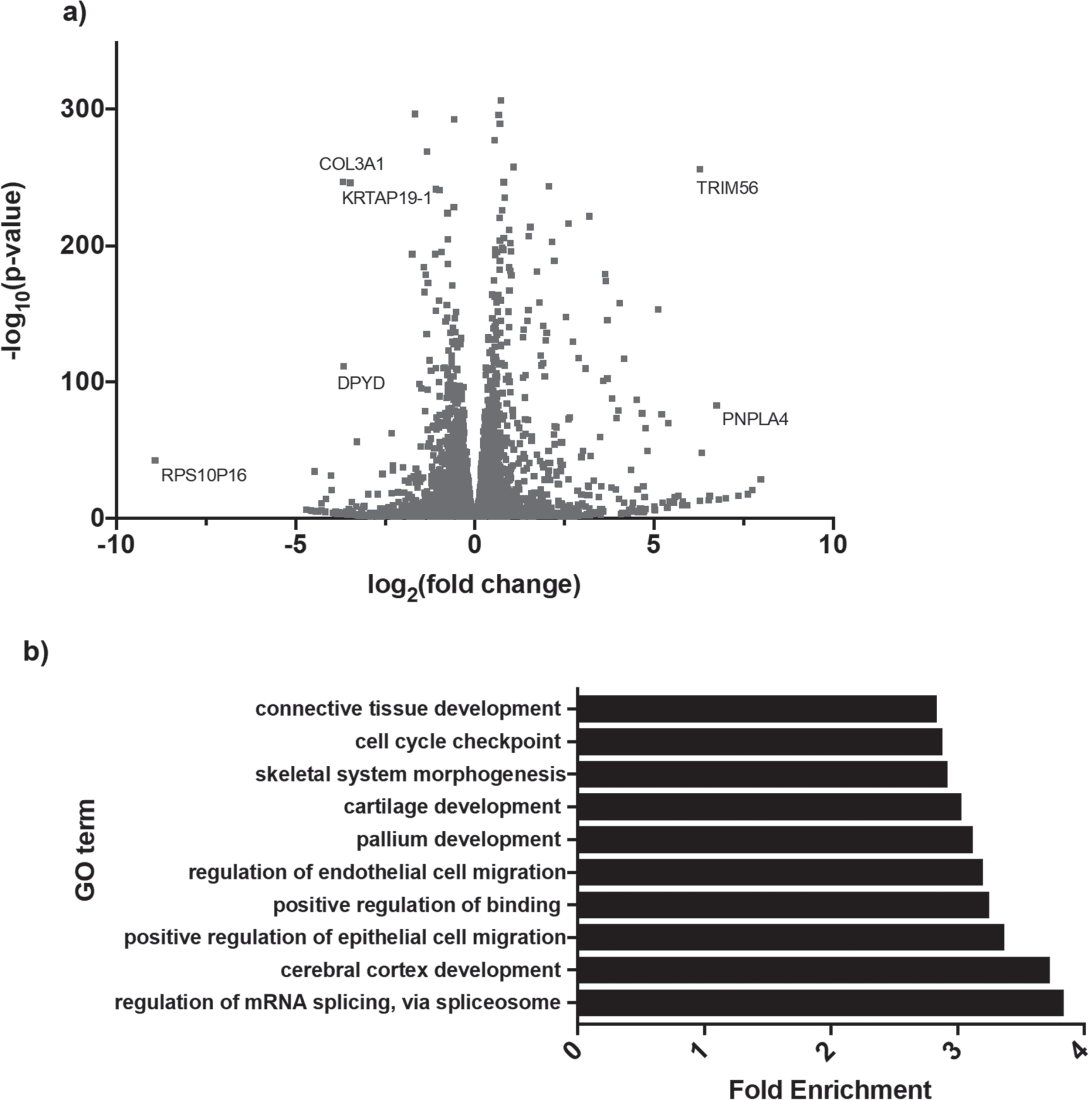
Transcriptome-wide differential gene expression in *EFTUD2*-knockdown cells. (**a**) Volcano plot showing all 10 442 significantly DEGs (*P*_adj_ < 0.05) in *EFTUD2*-knockdown cells obtained by whole transcriptome RNA-Seq and analysed using DESeq2. A total of 5323 DEGs were significantly downregulated (log_2_foldchange > 0) in 3.C9 cells while 5119 DEGs were significantly upregulated (log_2_foldchange < 0) in 3.C9 cells. RNA-Seq was performed on six replicate samples for each of WT and 3.C9 cells. **(b)** Top 10 enriched Gene Ontology (GO) terms for biological processes associated with the top 1000 most significantly (lowest p-value) differentially expressed genes in *EFTUD2*-knockdown cells, obtained using the PANTHER Overrepresentation Test (release 2019-03-08, annotation data set GO Biological Processes Complete, Fisher’s Exact test with Bonferroni correction).

### Widespread mis-expression of genes in *EFTUD2*-knockdown cells

We next used RNA-Seq to assess the effects of reduced *EFTUD2* expression on the transcriptome in 3.C9 cells and WT cells. Six replicate samples of total cellular RNA were extracted from WT cells and six replicate samples of total cellular RNA were extracted from 3.C9 cells grown under normal cell culture conditions and analysed by RNA-Seq.

Reduced *EFTUD2* expression in the 3.C9 cells resulted in the differential expression of 10 422 genes (both protein-coding and non-protein-coding) (*P*_adj_ < 0.05) (Fig. 4a). Approximately 51% (5323) of these differentially expressed genes (DEGs) were significantly downregulated in 3.C9 cells, while 49% (5119) of these were significantly upregulated in 3.C9 cells.

To determine whether particular subsets of genes were differentially expressed, we performed Gene Ontology (GO) analysis of the top 1000 most significant (lowest *P*-values) DEGs using the PANTHER Overrepresentation Test for enriched biological processes (49). Among the most enriched GO terms for biological processes were regulation of mRNA splicing via the spliceosome, cerebral cortex development, regulation of endothelial and epithelial cell migration, positive regulation of binding, pallium development, cartilage development and skeletal system morphogenesis (Fig. 4b). The prevalence of GO terms relating to development, in particular to development of the brain, cartilage and skeletal system, provides an immediate link between reduced *EFTUD2* expression and defective craniofacial development, as observed in patients with MFDGA. The enrichment of mRNA splicing regulation as a GO term supports several recent reports indicating that knocking down the expression of, or inhibiting, a spliceosomal protein affects the expression of other splicing factors (50,51). Interestingly, one downregulated gene in the *EFTUD2*-knockdown cell line was *TXNL4A*, the causative gene in Burn–McKeown Syndrome and another U5 snRNP protein (14,23,52). Western blot analysis confirmed that reduced *TXNL4A* mRNA expression led to significantly reduced TXNL4A protein in the 3.C9 cell line (Supplementary Material, Fig.S3), suggesting a potential link between the two U5 snRNP genes associated with spliceosomal craniofacial disorders.

Ingenuity Pathway Analysis (IPA) was then used to investigate the DEGs in terms of known upstream regulators and functional networks (53). Among the top upstream regulators identified were the ribosome biogenesis protein SBDS, the growth regulator OSM and TGFβ1 (Table 1). Interestingly, TGFβ signaling plays a critical role in craniofacial development, while loss of TGFβ signaling results in differentiation and cell–cell communication defects in cranial neural crest cells (NCCs) and is associated with syndromes that manifest with craniofacial malformations including Loeys–Dietz syndrome (54–57). The most highly enriched predicted molecular and cellular functions according to IPA were cell-to-cell signaling and interaction, cellular assembly and organization, cellular function and maintenance, cellular development and cellular growth and proliferation (Table 1). Indeed, all of these functions are known to be highly important in developmental processes. Furthermore, IPA identified several enriched functional networks associated with the DEGs, including networks associated with cell-to-cell signaling, cellular assembly and organization, cell cycle and embryonic development, cell death and survival, nervous system development and function and cell morphology, again revealing prevalence for networks vitally important in normal developmental processes (Table 1). Taken together, this analysis indicates mis-expression of genes leading to the widespread disruption of cellular processes but with an enrichment for processes involved in embryonic development when *EFTUD2* expression is reduced in human cells. In turn, such disruption may provide a link to the craniofacial and other developmental defects observed in MFDGA patients.

**Table 1:** Top five upstream regulators, molecular and cellular functions and functional networks associated with all significantly differentially expressed genes in *EFTUD2*-knockdown cells obtained using IPA

Top upstream regulators	Top molecular and cellular functions	Top associated networks
SBDS	Cell-to-cell signaling and interaction	Cell-to-cell signaling and interaction, cellular assembly and organization, cellular function and maintenance
OSM	Cellular assembly and organization	Cellular assembly and organization, cellular function and maintenance, molecular transport
TGFβ1	Cellular function and maintenance	Behaviour, hereditary disorder, neurological disease
Decitabine	Cellular development	Cancer, cardiovascular disease, cell-to-cell signaling and interaction
Dexamethasone	Cellular growth and proliferation	Cell cycle, developmental disorder, embryonic development

### Mis-splicing in *EFTUD2*-knockdown cells

To quantify and analyse the alterations in pre-mRNA splicing when *EFTUD2* expression is reduced in our human cell line model, we used the rMATS computational package to detect differential alternative splicing events including skipped exons (SE), the use of alternative 5′ or 3′ splice sites (A5SS and A3SS, respectively), mutually exclusive exons (MXEs) and retained introns (RIs) in our RNA-Seq data (58–60).

**Figure 5:**
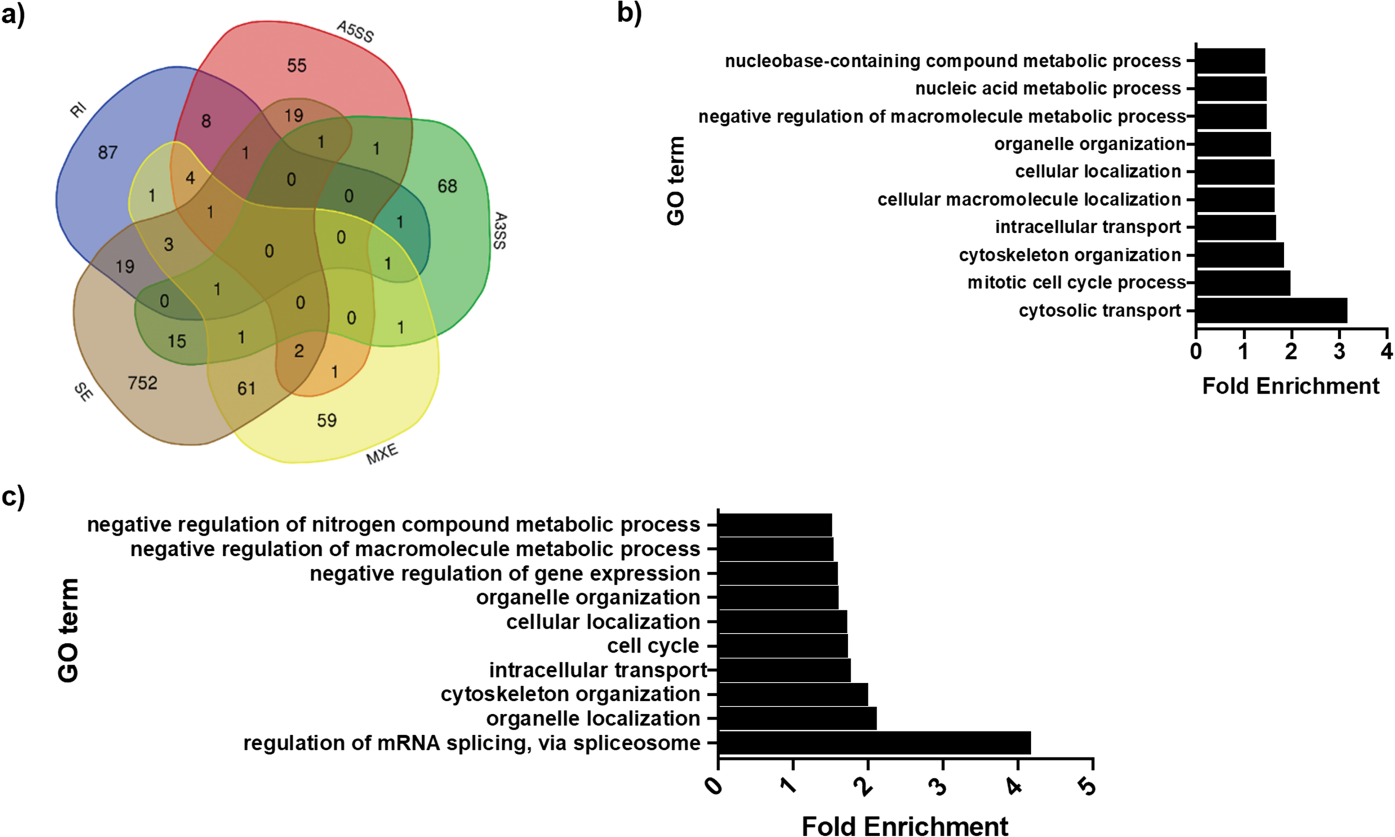
Global mis-splicing in *EFTUD2*-knockdown cells. (**a**) Venn diagram of numbers of genes displaying multiple forms of altered splicing in *EFTUD2*-knockdown cells. SE = skipped exon, RI = retained intron, MXE = mutually exclusive exons, A5SS = alternative 5′ splice site use, A3SS = alternative 3′ splice site use. (**b**) Top 10 enriched GO terms for biological processes associated with all genes displaying significantly altered splicing in *EFTUD2*-knockdown cells, obtained using the PANTHER Overrepresentation Test (release March 8, 2019, annotation data set GO Biological Processes Complete, Fisher’s Exact test with Bonferroni correction). (**c**) Top 10 enriched GO terms for biological processes associated with all genes displaying significantly altered exon skipping events in *EFTUD2*-knockdown cells, obtained using the PANTHER Overrepresentation Test release (March 8, 2019, annotation data set GO Biological Processes Complete, Fisher’s Exact test with Bonferroni correction).

In total, rMATS identified 1654 significant altered splicing events [*P* < 0.05 and false discovery rate (FDR) < 0.05] in 1161 different genes between the 3.C9 cells and WT cells, five of which were validated by RT-PCR (Supplementary Material, Fig. S4). We chose to validate altered splicing events in several categories (three SE, one RI and one MXE event) as these were some of the most significantly altered events (lowest *P*-values). Sashimi plots indicated sufficient read differences between 3.C9 cells and WT cells to detect by RT-PCR analysis (Supplementary Material, Fig. S5). There were 135 RI events in 125 different genes, of which 87 were also differentially expressed; 93 A5SS events in 93 genes, of which 61 were also differentially expressed; 97 A3SS events in 90 different genes, of which 61 were also differentially expressed; 199 MXE events in 136 genes, of which 102 were also differentially expressed; and 1130 SE events in 876 genes, of which 595 were also differentially expressed (Table 2). For the genes displaying both mis-splicing and differential expression, it is likely that the altered splicing is directly affecting the expression of the gene. A total of 142 genes displayed more than one significant altered splicing event, with two genes (*GAS5* and *RSRC2*) falling into four of the five categories of altered splicing events and 13 genes displaying three different forms of altered splicing between the WT cells and *EFTUD2*-knockdown cells (Fig. 5a; Supplementary Material, Table S1). It is not surprising that there is not a one-to-one relationship between mis-spliced genes and mis-expressed genes. For example, if a transcription factor responsible for regulating the expression of a number of downstream genes is mis-spliced, this may affect the expression of those downstream genes even though they are not themselves mis-spliced. Furthermore, mis-splicing does not necessarily alter the overall expression of a gene but may, for example, alter the balance of isoforms of mature transcripts (such as by altering how often a particular exon is included or skipped), which could in turn affect factors downstream of the original mis-spliced gene.

**Table 2:** Numbers of significantly altered exon skipping events, altered use of alternative 5′ and 3′ splice sites, altered mutually exclusive exon events and altered intron retention events in *EFTUD2*-knockdown cells

Class of alternative splicing	Number of events	Number of genes	Number of genes also differentially expressed
SE	1130	876	595
A5SS	93	93	61
A3SS	97	90	61
MXE	199	136	102
RI	135	125	87

The corresponding number of affected genes and the relationship between mis-splicing events and differential expression of affected genes was obtained through analysis of RNA-Seq data using rMATS v.4.0.2.

Here we found that SE is by far the most common mis-splicing event, accounting for >70% of the significant alternative splicing events. This is in agreement with data from other human models where the expression of a splicing factor is reduced or its activity inhibited, suggesting that human cells are particularly vulnerable to exon skipping events (50,61,62). However, in the zebrafish model described by Lei *et al.*, the authors identified that both SE and RI are equally prevalent when *eftud2* expression is knocked down in zebrafish (33). Of the 560 zebrafish genes in which SE was observed, 398 of these have orthologs in humans and 28 of these genes also displayed a significant exon skipping event in our 3.C9 cell line (Supplementary Material, Fig. S6).

To investigate whether particular subsets of genes are mis-spliced when *EFTUD2* expression is reduced, we next performed GO enrichment and IPA analysis on our 1161 mis-spliced gene dataset. The GO terms identified as most highly enriched included cytosolic transport, the mitotic cell cycle, cytoskeleton organization, cellular and organelle localization (Fig. 5b). Interestingly, one of the top predicted upstream regulators identified by IPA in the mis-spliced gene dataset was the apoptosis factor p53, while cell death and survival, cell cycle, neurological disease and organ morphology and developmental disorder networks were all identified as being enriched (Supplementary Material, Table S2). Regulated apoptosis is vitally important in craniofacial development, and aberrant apoptosis plays a role in other disorders with a craniofacial phenotype including Treacher Collins syndrome (17,63–67). Therefore, the association of mis-spliced genes in our human cell model with the upstream regulator p53 and the enrichment of cell death and survival networks suggest potential disruption of apoptotic pathways in *EFTUD2* haploinsufficient cells, which in turn may perturb normal craniofacial development.

We next focussed more specifically on genes in which significant SE events were identified, the only class of alternative splicing events with sufficient genes to permit reliable GO and IPA analysis. Of the 1130 total SE events, 623 (55%) showed increased exon skipping, while 507 (45%) showed decreased exon skipping in the 3.C9 cells compared to the WT cells. The most highly enriched GO terms associated with all the 876 genes in which SE was observed included regulation of mRNA splicing, organelle localization, cytoskeleton organization, intracellular transport and the cell cycle (Fig. 5c). The top associated upstream regulator identified by IPA analysis of SE genes was again p53, while enriched associated networks included developmental disorders, cell morphology, embryonic development, cell cycle, and cellular assembly and organization (Supplementary Material, Table S2). We then performed IPA analysis independently on the two classes of SE genes. For the genes in which SE events were upregulated in 3.C9 cells, the top upstream regulator identified was again p53, with a web of overlapping enriched networks including cell cycle, cellular assembly and organization, nucleic acid metabolism, gene expression and cellular/embryonic development (Supplementary Material, Table S2). In contrast, p53 was not a top enriched upstream regulator for the genes associated with downregulated exon skipping events in 3.C9; in this case, the sodium:neurotransmitter symporter SLC6A2 and the neuronal adaptor protein APBA1 were identified as top upstream regulators. Nonetheless, enriched networks in the group of genes with downregulated SE in 3.C9 cells included cell morphology, molecular transport, cellular assembly and organization, cellular function and maintenance and developmental disorders (Supplementary Material, Table S2). Therefore, the enrichment of exon skipping events in genes downstream of p53 appeared to be in genes in which exon skipping was increased in the *EFTUD2*-knockdown cells, but in both classes of SE genes (upregulated and downregulated) the networks identified by IPA analysis are highly linked to processes associated with cellular, tissue and organismal development.

### Features of SEs and RIs

Having investigated the functional relationships between mis-spliced genes, we turned our attention to why particular subsets and classes of genes are mis-spliced when *EFTUD2* expression is reduced. In particular, we focussed on the physical properties of exons that are significantly more or less skipped and introns that are significantly more or less retained in the *EFTUD2*-knockdown cells compared to the WT cells. For this analysis, each set of exons or introns was compared to two different control sets. For the RI analysis, controls were generated containing an intron plus its two flanking exons (E-I-E), both derived from the genes containing the RI but for introns that were not significantly retained (internal control introns), as well as from other genes in the genome that were identified as being highly expressed in the RNA-Seq data (external control introns). Highly expressed genes were selected to avoid cases of lowly expressed genes where higher expression levels would have revealed that there were altered splicing events, to prevent false positives. For the SEs, our controls were comprised of an exon, plus its two flanking introns and two flanking exons (E-I-E-I-E), again both derived from the genes containing the SE event but for an exon not significantly skipped (internal control exons) and exons from highly expressed genes outside of the DEG list (external control exons). The rationale behind these extended control sequences was that splicing is influenced by multiple sequences, both within the intron to be spliced out and the surrounding exons and introns.

**Figure 6:**
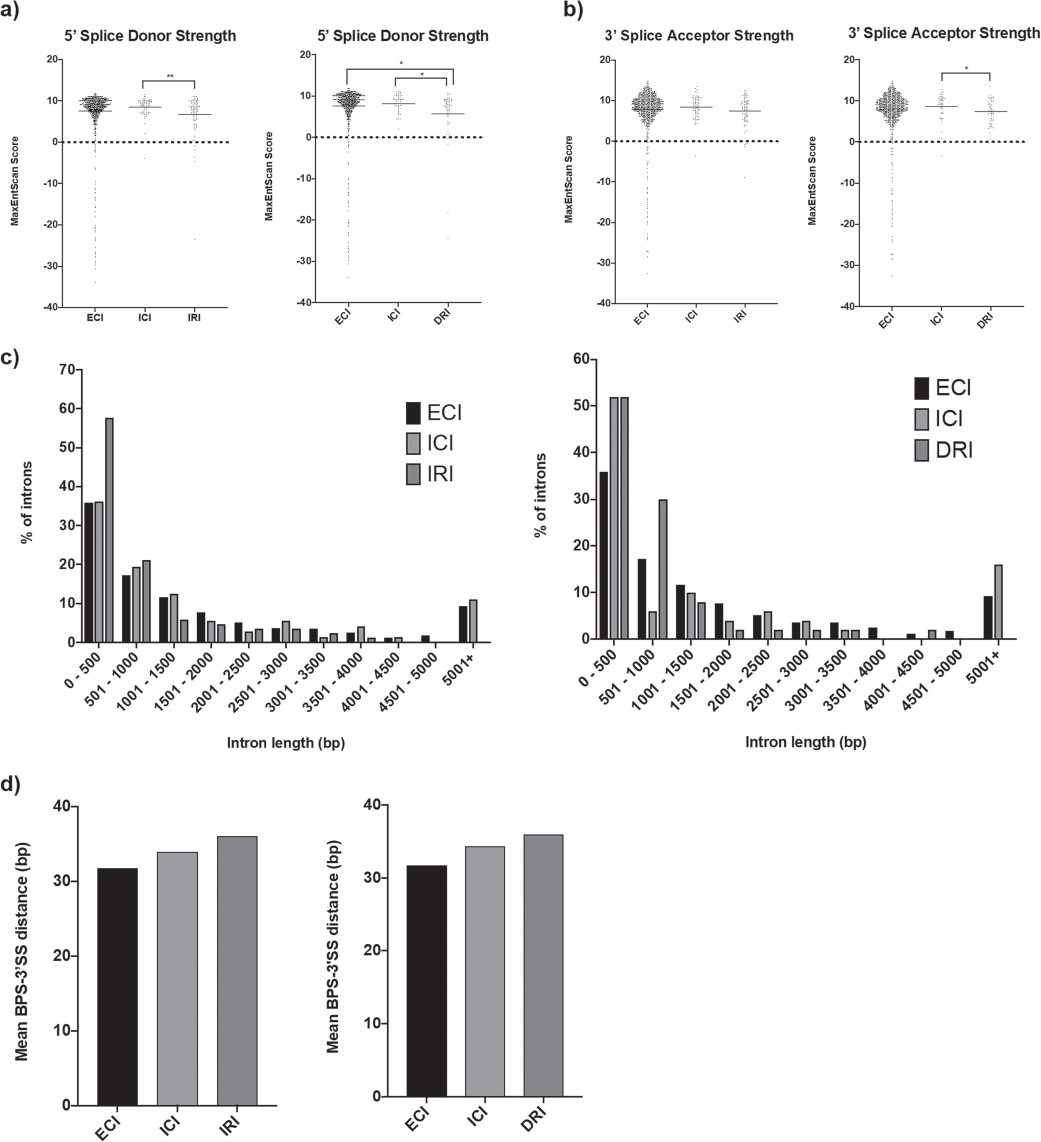
Characteristics of introns significantly more or less retained in *EFTUD2*-knockdown cells. (**a**) Strength of 5′ splice donor sequences for introns showing increased retention (IRI) and introns showing decreased retention (DRI) in 3.C9 cells, compared to external control intron (ECI) and internal control intron (ICI) splice donors, as obtained using MaxEntScan::score5ss. ^*^P-value < 0.05. ^**^P-value < 0.01. (**b**) Strength of 3′ splice acceptor sequences for IRI and DRI 3.C9 cells, compared to ECI and ICI splice donor sequences, as obtained using MaxEntScan::score3ss. ^*^P-value < 0.05. (**c**) Lengths of IRI and DRI in 3.C9 cells, compared to ECI and ICI. **(d)** Mean distance between predicted BPS and 3′ splice site (3′SS) in IRI and DRI in 3.C9 cells, compared to ECI and ICI, obtained using SVM-BPFinder.

For the RIs, we investigated the strength of the splice donor and acceptor sequences, the length and GC content of the RIs, the lengths of the flanking upstream and downstream exons, and the predicted BPS to 3′ splice site (BPS-3′SS) distance (Table 3; Supplementary Material, Fig. S7). There were no obvious differences in the GC content of the RIs (Supplementary Material, Fig. S7a) nor any differences in the lengths of the upstream and downstream exons of the RIs (Supplementary Material, Fig. S7b and c). However, we found that both introns that were significantly more retained and introns that were significantly less retained in 3.C9 cells had weaker 5′ splice donor sites than our intragenic control splice donor set. Additionally, in the case of splice donors for introns that were significantly less retained in 3.C9, these donor sites were also weaker than external control donors (Fig. 6a). Introns that were less retained in 3.C9 cells also had weaker 3′ splice acceptor sites than intragenic control acceptors, but there was no change in 3′ splice acceptor strength in introns more retained in 3.C9 cells (Fig. 6b). Furthermore, the introns that were more retained in 3.C9 cells were shorter, with 57% of the RIs shorter than 500 bp compared to 36% for both the external and internal control introns (Fig. 6c). For both classes of RIs the BPS-3′SS distance was slightly longer than the control sets (Fig. 6d). Our findings support the idea that *cis*-features of introns, including length and splice site strength, may be important determinants of intron retention, while also identifying the significance of the BPS-3′SS distance.

**Table 3:** Summary of the properties of introns significantly more or less retained in 3.C9 cells

Property	Introns more retained in 3.C9 cells	Introns less retained in 3.C9 cells
Splice donor strength	Weaker than internal control donors	Weaker than internal and external control donors
Splice acceptor strength	No significant difference	Weaker than internal control acceptors
Intron length	Shorter	No obvious differences
Intron GC content	No obvious differences	No obvious differences
Length of upstream exon	No obvious differences	No obvious differences
Length of downstream exon	No obvious differences	No obvious differences
Predicted BPS-3’SS distance	Longer	Longer
% of RIs with in-frame stop codon	93%	86%

To investigate whether particular sequences were associated with the RIs, we next scanned our RI plus flanking exon sequences for enriched 5–20 nt sequence motifs using the MEME suite software, using our internal and external controls as background sets independently (68–70). We found several enriched motifs for both introns subsets (retained more, or less retained in 3.C9 cells), suggesting that the introns susceptible to altered retention when *EFTUD2* expression is reduced share common sequence features (Supplementary Material, Table S3). Interestingly, for both classes of RIs, the motifs occurred in both surrounding exon and RI sequences, suggesting that position of the motif is not critical. All the RI E-I-E sequences contained at least one motif in each case, and there was no correlation between the number of motifs a RI is associated with and the degree of intron retention (Supplementary Material, Fig. S7d and e). If and how these enriched motifs contribute to mis-splicing in the *EFTUD2*-knockdown cells is unclear, although one possibility is that they represent binding sites for particular RNA-binding proteins. Indeed, scanning the RNA-binding protein database (RBPDB) identified potential binding sites for several known proteins, including FUS, MBNL1, RBMX, KHSRP, SFRS1 and SFRS9, within these motifs. Interestingly, using the two different control sets yielded different enriched motifs for each class of RI.

To assess the impact of intron retention on gene output, we next looked for the presence of in-frame stop codons in the RIs for both classes of RIs. For the introns with increased retention in 3.C9 cells, the majority (93%) contained an in-frame stop codon, while 2% generated a frameshift creating a premature stop codon in the immediately downstream exon (Table 3). In these cases, the retention of the intron would likely reduce overall protein output from the affected gene by NMD. However, it should be noted that levels of NMD may not be equivalent across all genes; the position of the premature stop codon may play a role, as may the presence of internal ribosome entry sites (71, 72). For the introns retained less frequently in the *EFTUD2*-knockdown cells, 86% contained in-frame stop codons while 4% generated a frameshift; for these genes, the protein output is likely increased in the 3.C9 cells versus WT cells (Table 3). Furthermore, it can be postulated that those RIs that do not contain in-frame stop codons and do not generate a shift in the reading frame with the creation of a premature stop codon are potentially functional introns.

**Figure 7:**
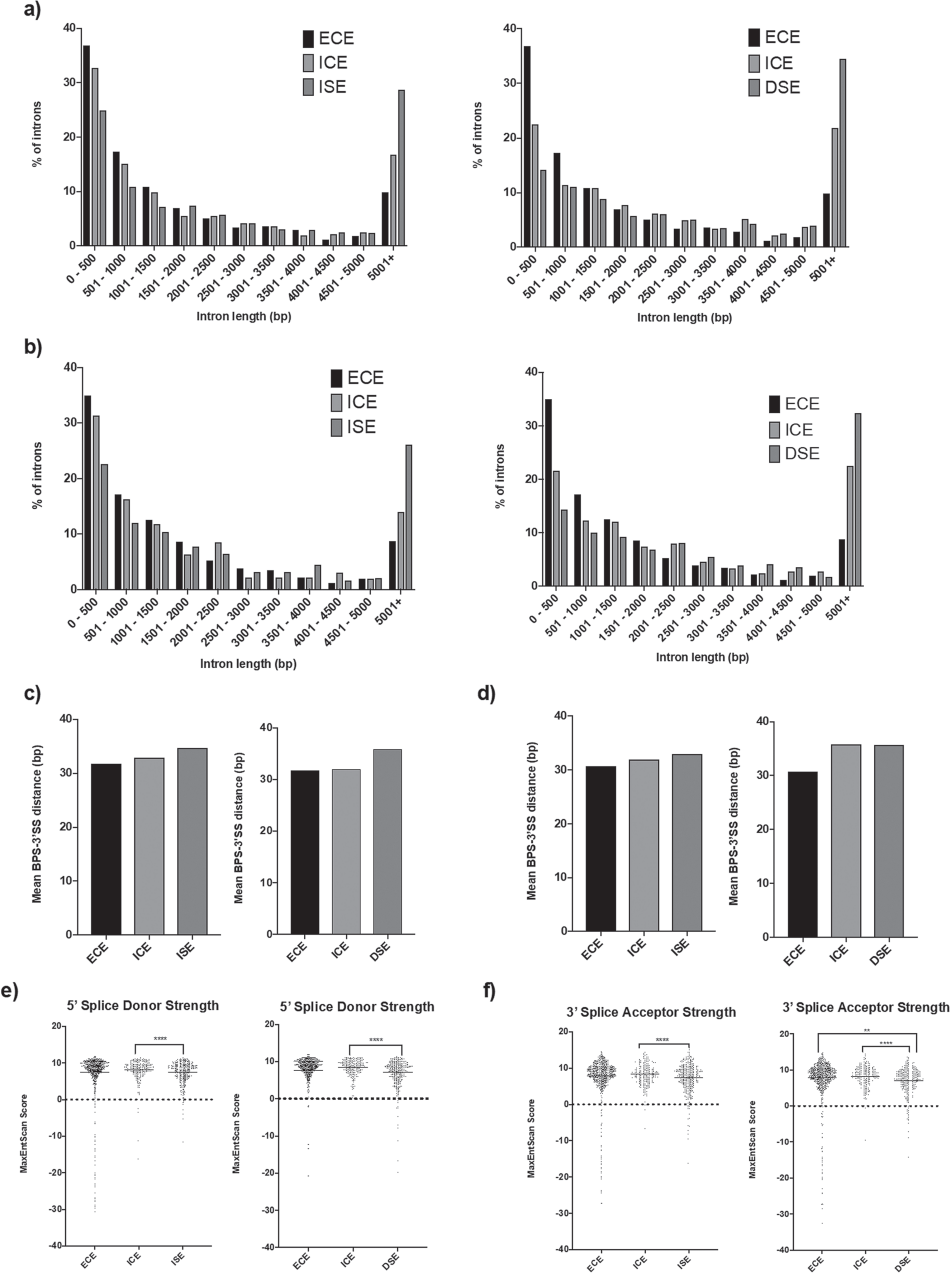
Characteristics of exons displaying significantly increased or decreased skipping in 3.C 9 cells. (**a**) Lengths of immediately upstream introns for exons showing increased skipping (ISE) and exons showing decreased skipping (DSE) in 3.C9 cells, compared to external control exons (ECE) and internal control exons (ICE). (**b**) Lengths of immediately downstream introns for ISE and DSE in 3.C9 cells, compared to ECE and ICE. (**c**) Mean distance between predicted BPS and 3′ splice site (3′SS) in introns immediately upstream of ISE and DSE in 3.C9 cells, compared to ECE and ICE, as obtained using SVM-BPFinder. (**d**) Mean distance between predicted BPS and 3′SS in introns immediately downstream of ISE and DSE in 3.C9 cells, compared to ECE and ICE, as obtained using SVM-BPFinder. (**e**) Strength of 5′ splice donor sequences for ISE and DSE in 3.C9 cells, compared to ECE and ICE splice donor sequences, as obtained using MaxEntScan::score5ss. ^****^P-value < 0.0001. (**f**) Strength of 3′ splice acceptor sequences for ISE and DSE in 3.C9 cells, compared to ECE and ICE splice acceptor sequences, as obtained using MaxEntScan::score3ss. ^**^P-value < 0.01, ^****^P-value < 0.0001.

For the SEs, we investigated the splice donor and splice acceptor strengths for the SE itself, the strengths of the upstream splice donor and the downstream splice acceptor, the lengths of the SE and the two flanking introns, the GC content of the SEs and the two flanking introns and the BPS-3′SS distances for the upstream and downstream flanking introns (Table 4; Supplementary Material, Fig. S8). While there were no obvious differences in exon length or exon GC content compared to either set of controls (Supplementary Material, Fig. S8a and b), the SEs were associated with longer upstream and downstream introns, although the GC content of these flanking introns was no different (Fig. 7a and b; Supplementary Material, Fig. S8c and d). Furthermore, the upstream introns were associated with slightly longer BPS-3′SS distances for both types of differentially expressed exons (more or less skipped) in 3.C9 cells (Fig. 7c). For exons with increased skipping in 3.C9 cells, the downstream intron BPS-3′SS distance was longer than both external and internal controls; although for exons with reduced skipping in 3.C9 cells, the downstream BPS-3′SS distance was only longer than the external controls (Fig. 7d). We found that exons both more and less skipped in 3.C9 cells were associated with weaker splice donor and splice acceptor sites compared to intragenic control donor and acceptor splice sites respectively (Fig. 7e and f). For the exons skipped more in 3.C9 cells, there were no apparent differences in the strength of the upstream splice donors compared to either control set, while the downstream splice acceptors were weaker than the external control set but not relative to intragenic control downstream splice acceptors. In contrast, exons that were skipped less in the 3.C9 cells than WT cells were associated with stronger upstream donors than internal control upstream donors, and weaker downstream splice acceptors compared to both sets of controls (Supplementary Material, Fig. S8e and f).

**Table 4:** Summary of the properties of exons significantly more or less skipped in 3.C9 cells

Property	Exons more skipped in 3.C9 cells	Exons less skipped in 3.C9 cells
Splice donor strength	Weaker than internal control donors	Weaker than internal control donors
Splice acceptor strength	Weaker than internal control acceptors	Weaker than both external and internal control acceptors
Upstream splice donor strength	No significant differences	Stronger than internal control upstream donors
Downstream splice acceptor strength	Weaker than external control downstream acceptors	Weaker than internal and external control downstream acceptors
SE length	No obvious differences	No obvious differences
SE GC content	No obvious differences	No obvious differences
Upstream intron BPS-3′SS distance	Longer	Longer
Downstream intron BPS-3′SS distance	Longer	Longer than external controls only
Upstream intron length	Longer	Longer
Downstream intron length	Longer	Longer
Upstream intron GC content	No obvious differences	No obvious differences
Downstream intron GC content	No obvious differences	No obvious differences

We next scanned our SE E-I-E-I-E sequences for enriched 5–20 nt sequence motifs, using both our internal and external controls as background sets. No enriched motifs were identified in exons that were skipped less in the *EFTUD2* mutant cells, but nine enriched motifs were identified for the exons skipped more in 3.C9 cells when using the internal control set as a background and three enriched motifs were identified when using the external controls, with a degree of overlap between the two groups of enriched motifs (Supplementary Table S4). The number of motifs associated with the E-I-E-I-E sequences ranged from zero to nine (internal control set) or zero to four (external control set), and there was no obvious relationship between number of motifs and degree of exon skipping (Supplementary Material, Fig. S8g). Again, it can be postulated that the enriched sequence motifs may serve as binding sites for binding proteins, and indeed putative binding sites for proteins including RBMX, MBNL1, RBM4, FUS and SFRS1 were identified in the enriched motifs discovered using the internal controls while sites for SFRS9, SFRS1, FUS and RBM4 were identified in the enriched motifs using the external controls.

## Discussion

How variants in core pre-mRNA splicing factors required for splicing of all pre-mRNAs result in distinct craniofacial defects is still largely unknown. Here we present the first human cell model of MFDGA to study the effects of reduced *EFTUD2* expression on human cell function. Reducing *EFTUD2* expression negatively affects cell proliferation with cells being particularly sensitive to conditions that induce ER stress and displaying differential expression of several genes involved in the ER stress response and NMD pathway. Furthermore, there is transcriptome-wide mis-expression and mis-splicing of genes, with a particular tendency for exon skipping events, although intron retention, alternative 3′ and 5′ splice sites and MXEs are also observed. This prevalence of exon skipping events is in agreement with previous reports noting high levels of exon skipping when other core spliceosome factors are inhibited or knocked down in human cells (50,61,62). In contrast, an *eftud2*-knockdown zebrafish model revealed equally large numbers of both SE and intron-retaining transcripts (33). Thus, it appears that human cells are particularly vulnerable to exon skipping events when the expression of spliceosome components is reduced, but why this is the case is not clear. Overall, we show that in *EFTUD2*-knockdown human cells there is mis-splicing of a subset of pre-mRNAs which share common *cis*-features, and this mis-splicing particularly affects genes important in developmental processes, including craniofacial development, providing a clear link to the craniofacial defects observed in patients with MFDGA.

Interestingly, in the zebrafish model increased apoptosis and altered mitosis of neural progenitors suggested a role for *eftud2* in the survival and differentiation of neural progenitors (33). The authors proposed that aberrantly spliced transcripts overburdened the NMD pathway and activated p53-dependent apoptosis (33). Our work expands upon these findings as mis-expression and/or mis-splicing of NMD, UPR and ER stress response genes seen here would compromise these pathways contributing to the inability of the NMD pathway to overcome effectively the increased burden on the ER stress response, thereby promoting increased p53-dependent apoptosis. Why cells of the developing craniofacial region are particularly affected remains to be fully understood. However, enrichment of *eftud2* in zebrafish embryo neural progenitors, highly concentrated *eftud2* expression in the brain of zebrafish embryos 36 h post-fertilization and enrichment of *Eftud2* in the mouse ventricular forebrain suggests neural progenitors are particularly dependent on EFTUD2 (32,33,73). Neural progenitors also have a very high turnover of pre-mRNAs and high levels of alternative splicing, suggesting an increased burden on the spliceosome compared to other tissues (74–77). Furthermore, NCCs, the key cell population responsible for development of the vertebrate head, are particularly sensitized to activated p53 and are 2-fold more likely to undergo apoptosis when exposed to stabilized p53 (63,64). In fact, NCCs express higher levels of p53 than other cell types during normal development (63,65). Taken together, our work supports a model where mis-splicing and/or mis-expression of key ER stress response genes renders cells unable to manage the burden of aberrantly spliced transcripts, triggering p53-dependent cell death in cells of the developing craniofacial region. Indeed, aberrant cell death during development leads to craniofacial defects in Treacher Collins syndrome while ER stress-induced apoptosis is implicated in several neurological diseases, including Alzheimer’s and Parkinson’s disease (67,78–81).

Analysis of functional and physical properties of genes mis-expressed and/or mis-spliced in *EFTUD2*-knockdown cells has revealed how reduced *EFTUD2* expression might cause craniofacial defects. The enrichment of GO terms including cerebral cortex development, cartilage development, skeletal system morphogenesis and connective tissue development in DEGs clearly indicates that genes involved in pathways relating to development, particularly of the brain and craniofacial region, are directly affected by reduced *EFTUD2* expression. Furthermore, identification of TGFβ1 as a top enriched upstream regulator of the mis-expressed genes is interesting, given the known importance of TGFβ signaling in craniofacial development and the association of defects in TGFβ signaling with cleft palate, a common phenotype observed in MFDGA patients (55,56). The identification of p53 as a top upstream regulator (in particular for the genes with increased exon skipping in 3.C9 cells) provides yet another link between reduced *EFTUD2* expression and the apoptosis pathway. Additionally, the prevalence among mis-spliced genes of networks associated with cell death, cellular assembly and organization, cell signaling, organ morphology and embryonic development again implicates altered *EFTUD2* expression in processes linked to development. It is worth noting that the most highly enriched GO term associated with the DEGs and SE genes is mRNA splicing regulation via the spliceosome suggesting a co-regulated network of splicing factor expression (50,51). Whether this represents an ability of the spliceosome to adjust to fluctuations to maintain homeostasis of its various components remains to be seen, although this is certainly the case with other macromolecular machines such as the ribosome (82).

Analysis of SE and RI properties has helped characterize features that are particularly sensitive to *EFTUD2* expression changes. Our findings suggest that particular subsets of introns and exons, with common physical and sequence properties, are mis-spliced when *EFTUD2* expression is reduced. Our finding that the RIs have distinct splice site strength, length and BPS-3′SS distance characteristics, as well as sharing several enriched sequence motifs, agrees with previous work documenting *cis*-features of physiologically RIs including shorter length, weaker splice sites and higher GC content (33,83,84). While we did not observe differences in GC content, our work has indicated the significance of the BPS-3′SS distance. Work in yeast has demonstrated that branch site repositioning between the two steps of splicing is more difficult for transcripts with longer BPS-3′SS and can be influenced by secondary structure (85). Our observation that both classes of RIs contained longer BPS-3′SS distances agrees with these findings. How and why introns with the particular *cis*-features identified here are particularly affected by reduced *EFTUD2* levels remains to be seen, although we postulate that the short length, weaker splice sites and long BPS-3′SS make certain introns harder to splice and reduced levels of a core spliceosome protein is sufficient to ‘tip’ the balance inducing mis-splicing. Furthermore, identification of several enriched motifs in and around RIs suggests that certain sequence characteristics render particular introns more vulnerable to changes in *EFTUD2* expression, possibly through the binding of auxiliary proteins such as RBMX and MBNL1. Particularly intriguing are the genes where an intron is less retained, implying reduced levels of *EFTUD2* improve splicing efficiency. How reducing core spliceosome protein levels could improve splicing is still an open question, but it is possible that an intron retention serves a physiological function (such as modulating gene output through creation of PTCs and NMD) so reduced intron retention has potential to disrupt cellular processes in the same way as increased intron retention.

Our findings that exons that are more or less skipped are associated with significantly different splice site strengths, longer upstream and downstream intron BPS-3′SS distances and longer upstream and downstream introns, again suggest that distinct subsets of exons sharing common features are more susceptible to altered skipping when *EFTUD2* is knocked down. Previous work has shown a correlation between increasing upstream BPS-3′SS distance and exon skipping events in mammalian cells and between increased length of flanking introns and exon skipping (79,80). It is intriguing that enriched sequence motifs (which tend to be located in intronic *Alu* elements proximal to the exon) were only identified for exons skipped more in 3.C9 cells and not for exons skipped less. There have been previous reports showing that the insertion of a polymorphic *Alu* sequence into intronic regions close to an alternatively spliced exon promotes skipping of that exon (86). Future work should investigate whether these enriched sequences play a role in promoting exon skipping events when *EFTUD2* expression is reduced and whether the proximal *Alu* element contributes to exon skipping. Previous findings have reported common sequence motifs with base-pairing potential in exons known to undergo physiological alternative exon skipping (81). Additionally, several putative RNA-binding protein sites were identified in the SE enriched sequences*,* and disruption of RNA-binding proteins interactions with pre-mRNA promotes exon skipping in other human disorders (87). Interestingly, histone modifications and epigenetic markers are important for exon skipping (88). If reduced expression of *EFTUD2* affects histone modifying genes, skipping of exons in other genes could be affected indirectly. The complexity of human splicing and its regulation makes extracting direct versus indirect effects of reduced splicing factor expression all the more difficult.

Models of other craniofacial disorders have been developed to determine how variants in core splicing and EJC factors cause disease. For example, haploinsufficiency of core EJC components, including *EIF4A3*, cause microcephaly by converging regulation of p53 signaling (89,90). Haploinsufficiency of *EIF4A3* phenocopies the aberrant neurogenesis and microcephaly seen in patients, with downregulation of p53 rescuing the microcephaly. In models of RCPS, iPSCs and mouse models with *EIF4A3* deficiency revealed that EIF4A3 is required for NCC migration and osteochondrogenic differentiation during craniofacial development (91). Reduced expression of the U2 snRNP gene *SF3B4*, found in Rodriguez Acrofacial Dysostosis, disrupted growth plate architecture and led to reduced expression of *DLX5*, *DLX6*, *SOX9* and *SOX6* transcription factor genes, known to be important for skeletal development, in growth plate chondrocytes. The reduced expression of *SF3B4* also caused defects in pre-mRNA splicing, in particular aberrant exon skipping, similar to our *EFTUD2* haploinsufficient cells (61). Finally, *SF3B4* knockdown in Xenopus embryos, in a model for Nager syndrome, revealed that *SF3B4* depletion reduced expression of neural crest genes *sox10*, *snail2* and *twist* at the neural plate border, which was associated with neural plate broadening (92). In this Nager syndrome model there was also a reduced number of NCCs, and hypoplasia of neural crest-derived craniofacial cartilages was observed (92). Overall, these models have begun to decipher the developmental and splicing pathways that may be disrupted to cause specific disease phenotypes. In the future, developing a more complex NCC disease model derived from patient-specific iPSCs for MFDGA should provide more insight into how mutations in *EFTUD2* affect the key cells involved in craniofacial development. Additionally, MFDGA mouse models would allow investigation of the effects of reduced *EFTUD2* expression at the correct developmental stage *in vivo*.

Another question that remains to be addressed is why variants in different components of the same core spliceosome complex cause distinct tissue-specific diseases. For example, why variants in the U5 snRNP genes *PRPF6*, *PRPF8* and *SNRNP200* are associated with retinal degeneration in adRP, while variants in the U5 snRNP genes *EFTUD2* and *TXNL4A* are linked to craniofacial disorders, is not understood. However, the report of *CWC27* variants associated with both craniofacial and retinal defects indicates that overlap is possible (13,16). Indeed, it would be interesting to determine whether MFDGA patients develop retinal problems later in life. Spliceosomal genes associated with craniofacial disorders may be functionally linked in the spliceosome, and disruption of this functional network may be responsible for mis-splicing of critical genes involved in craniofacial development. For example, the recent cryo-EM structure of the human U4/U6.U5 tri-snRNP has revealed that TXNL4A is linked with EFTUD2 via PRPF8 and the U5 snRNA loop I (28,93). Furthermore, an interface between EFTUD2 and EIF4A3 has recently been identified and may connect the EJC with the spliceosome (94). Additionally, SF3B4 and EFTUD2 have been reported to interact and this interaction promotes recruitment of the tri-snRNP to the spliceosome via the U2 snRNP (95). Finally, the Sm ring of proteins binds to the U5 snRNA adjacent to the binding site for EFTUD2, providing a basis for a potential interaction between EFTUD2 and SNRPB (the affected protein in CCMS) (94). Thus, physical and functional links between the spliceosomal proteins associated with craniofacial disorders have been established. While our data have provided insight into how disruption of these functional links affects the splicing of just a subset of pre-mRNAs and why this specifically affects craniofacial development, the exact details of how these functional links specifically affect splicing remains to be determined.

## Materials and Methods

### Cell culture and treatments

HEK293 cells were cultured in 1X Dulbecco’s Modified Eagle Medium (DMEM) (Sigma) supplemented with 10% fetal bovine serum (FBS) (Sigma) and incubated in 5% CO_2_ at 37°C, unless otherwise stated. DTT, Tun and NaCl were added to the culture medium at the stated concentration and cells incubated under normal culture conditions for the stated durations following treatment. Original HEK293 cell stocks were supplied at passage 19, and experiments were not conducted on cells at passage numbers greater than 30.

### CRISPR-Cas9 nickase strategy for generating *EFTUD2* mutant cell lines

For the design of guide RNA (gRNA) targets for the high specificity CRISPR-Cas9 nickase strategy, CHOPCHOP software (http://chopchop.cbu.uib.no/) was utilized to identify candidate 20 nt target sequences adjacent to a PAM (5′NGG) sequence within *EFTUD2* (96, 97). Two Cas9n target sites on opposite DNA strands were selected to generate a pair of nicks that would result in site-specific double strand breaks and non-homologous end joining, whereas any off-target individual nicks would be repaired by the high-fidelity base excision repair pathway. gRNA constructs were generated by cloning phosphorylated primers (Supplementary Primer Table 1) for gRNA target DNA sequences into the *BbsI* sites of the pX335-U6-Chimeric_BB-CBh-hSpCas9n (D10A) (Addgene 42 335) vector (34). All constructs were sequenced to verify correct gRNA-containing constructs. HEK293 cells (passage 21) were seeded at a density of 2.5 × 10^5^ cells per well in a six-well plate, cultured to 40–60% confluency and transfected with the gRNA constructs using Lipofectamine 2000 (Invitrogen) according to the manufacturer’s instructions and incubated for 48 h. After 48 h, the transfected cells were detached from the plates with trypsin/EDTA (Sigma) and diluted before seeding into a 96-well plate to give an approximate density of one cell per well. Media was changed after 1 week and wells containing single colonies derived from a single cell were identified. Two weeks after transferring to a 96-well plate, mutant clones were screened by PCR of genomic DNA. For mutant screening, cells were washed with phosphate-buffered saline (PBS) (Sigma) and trypsin/EDTA was used to generate a cell suspension. A portion of this suspension was seeded in 6-well cell culture plates for propagation of the clones, while 50 μl was reserved for genomic DNA extraction. Genomic DNA was extracted using the Qiagen QIAamp DNA Mini Kit. Gene mutants were identified by PCR of the genomic DNA using primers specific for the flanking regions of the genes targeted by the gRNAs (Supplementary Primer Table 2).

To sequence the exact mutations introduced by the CRISPR/Cas9 nickase method the confirmation primers were designed to include *EcoRI* and *KpnI* cleavage sites (Supplementary Primer Table 3). Genomic DNA from mutant candidates was amplified by PCR, separated on an agarose gel and the PCR products purified using the Qiagen QIAquick Gel Extraction Kit. The purified PCR products were cloned into pBluescript II SK+ plasmid and the resulting plasmids sequenced.

### Western blot analysis

Cells were seeded at a density of 2.0 × 10^6^ cells in a 100 mm cell culture dish and grown to ~90% confluency. Total protein lysates were extracted using RIPA lysis buffer (Sigma), supplemented with a protease inhibitor cocktail covering a wide range of target proteases (Promega G6521). Protein concentration of the samples was determined using Bradford Reagent (Sigma-Aldrich) according to manufacturer’s recommendations, using bovine serum albumin (BSA) as a protein standard.

Cell lysates were denatured by boiling in SDS loading dye for 5 min and then separated by SDS-PAGE and transferred to nitrocellulose membrane using a BioRad TransBlot SD. Membranes were blocked with PBS (Sigma) + 5% non-fat dry milk and then incubated with primary antibodies (alpha-tubulin Sigma T9026 1:20000; EFTUD2/Snu114 custom-made rabbit antibody 1:10000) (98). Detection was performed using a LI-COR Odyssey system, with IRDye 800CW secondary antibodies (IRDye 800CW goat anti-mouse IgG 925–32 210, 1:5000; IRDye 800CW goat anti-rabbit IgG 925–32 211, 1:5000). Image quantification was performed using the LI-COR Image Studio software.

### Cell counting

Cells were plated in a six-well culture dish at a density of 2.5 × 10^5^ cells per well, indicated reagents added to culture medium at the specified concentration if applicable, and cells incubated for 48 h in 5% CO_2_ at 37°C. Cells were put into suspension using trypsin/EDTA treatment of the adherent cells, and DAPI (1 mg/ml) (Invitrogen) and Acridine Orange (800 μg/ml) (Invitrogen) were added to a final dilution of 1:100. Then 20 μl of the final mixture was loaded into a Chemometec NC-Slide A8 (Chemometec). Cell counting assays were performed using a NC250 Automated Cell Analyser Nucleocounter (Chemometec), and analysis performed using Nucleoview NC-250 software.

### Detection of proliferating cells

HEK293 WT and 3.C9 cells cultured on coverslips were fixed with 4% paraformaldehyde then permeabilized with 0.1% Triton X-100 in PBS (PBT). The cells were then blocked using 3% BSA in 0.2% Tween PBS solution. This blocking was followed by overnight incubation with primary anti-phospho-Histone H3 (Ser10) (Millipore 06-570, 1:200) diluted in blocking buffer. Cells were washed with PBT followed by incubation with goat anti rabbit Alexa Fluor 568 (1500) secondary antibody for 1 h. Cells were washed with PBT followed by DAPI nuclear stain and mounting. Three replicate coverslips for each cell line were analysed. Five images at 63× magnification of each replicate coverslip (15 total images) were used for counting percentage of cells with positive histone H3 phospho-Ser10 (% proliferating cells) or for counting the number of proliferating cells with rosette chromosome conformation. ImageJ was used for image analysis.

### RNA extraction

Total RNA was extracted from cells with TRIzol reagent (Invitrogen) according to the manufacturer’s instructions. The resulting RNA was cleaned up using the Qiagen RNeasy Mini RNA Isolation Kit, including a DNA digestion step. The RNA was either used immediately to synthesize cDNA or stored at −80°C.

### cDNA synthesis

Total RNA was converted into cDNA using Superscript IV (Invitrogen) and random hexamers (Invitrogen) according to the manufacturer’s protocol. cDNA was either used immediately as a template for quantitative or reverse transcription PCR or stored at −20°C.

### qPCR

qPCR reactions were performed with 2X PowerUp SYBR Green PCR Master Mix (Invitrogen). Primer pairs were retrieved from Primer Bank (http://pga.mgh.harvard.edu/primerbank) and are listed in Supplementary Primer Table 4 (99, 100). Fluorescence was detected using the StepOnePlus Real-Time PCR System (Applied Biosystems) in a 96-well plate format, using the standard Comparative C_T_ reaction cycle programme and associated data analysis software. Gene expression was assessed using the $\Delta \Delta$C_T_ method, using *ACTB* as a stable endogenous control. *ACTB* was chosen as the endogenous control gene because the raw C_T_ values were not significantly different between WT and 3.C9 cells, *ACTB* expression was not significantly different between the two cell lines in RNA-Seq data and the C_T_ values were similar to the C_T_ values for the test genes (101). Other endogenous control genes tested (*GAPDH*, *RPL13A*, *PPIA* and several core spliceosomal factors) did not fulfil one or more of these criteria (data not shown). Specificity of qPCR primers was confirmed by a single peak in melt curve analysis included in the standard reaction cycle programme.

### RT-PCR

RT-PCR reactions were performed with Phusion High-Fidelity DNA Polymerase according to manufacturer’s recommendations, using cDNA as a template. Primer pairs were designed using Primer-BLAST (www.ncbi.nlm.nih.gov/tools/primer-blast) and are listed in Supplementary Primer Table 4 (102).

PCR products were separated by electrophoresis on agarose gels, using SafeView nucleic acid stain (NBS Biologicals) and imaging on a UV transilluminator to reveal the bands. New England Biolabs Low Molecular Weight DNA Marker was loaded as a DNA size standard. Where stated, RT-PCR products were purified using the Qiagen QIAquick Gel Extraction Kit and identities confirmed by sequencing.

### RNA-Seq

Total cellular RNA was extracted as above from cells seeded at a density of 2.5 × 10^5^ cells per well in a six-well cell culture plate and grown until approximately 80% confluent. One microgram of RNA from each sample was diluted into 20 μl nuclease-free water, and quality and integrity of the RNA samples assessed using a 2200 TapeStation (Agilent Technologies). A poly-A enrichment library was then generated using the TruSeq® Stranded mRNA assay (Illumina, Inc.), according to the manufacturer’s protocol, and 76 bp (+ indices) paired-end sequencing carried out on an Illumina HiSeq 4000 instrument to median depths of 100 000 994 reads (WT; 76 921 284–125 079 436 reads; *n* = 6) and 111 361 009 reads (3.C9; 83 297 932–286 166 683 reads; *n* = 6). Output data were de-multiplexed (allowing one mismatch) and BCL-to-Fastq conversion performed using Illumina’s bcl2fastq software, version 2.17.1.14. Low quality bases and adapter sequences were trimmed using Trimmomatic, and reads aligned to the GRCh38 genome using the single-pass mode of the STAR aligner (v2.5.3a), as well as to the transcriptome according to the Gencode v28 human gene annotation (downloaded from https://www.gencodegenes.org/human/release_28.html) (103, 104). Differential expression analysis was conducted using the Bioconductor DESeq2 package v1.20.0 using default parameters (105). rMATS v.4.0.2 was used to identify mis-splicing events (58–60). Results were filtered to retain only events with a *P*-value < 0.05 and FDR < 0.05. For each gene showing a RI or SE event, we generated control sequences from elsewhere within the gene (‘internal’ controls) of either exon-intron-exon (E-I-E; for RIs) or exon-intron-exon-intron-exon (E-I-E-I-E; for SEs). These were generated randomly for each gene from the longest protein-coding transcript with transcript support level in the Gencode v28 annotation (where protein-coding transcripts were present); these sequences could not overlap the loci of any of the corresponding event E-I-E/E-I-E-I-Es from the original rMATS data, making construct generation impossible for some transcripts with low exon number. A single construct was chosen randomly from the pool of valid constructs for each transcript to make the final control sets. To generate external control sequence constructs, expression analysis was first carried out with RSEM v1.3.0 using default parameters (106). The 1200 most expressed genes that were not located on the mitochondrial genome nor present in any of the five filtered rMATS datasets were selected. We then selected the optimal transcript for each gene (as for the internal controls) and generated random E-I-E-I-E constructs for each transcript, provided there were at least three exons in the gene. GO analysis was performed using the PANTHER Overrepresentation Test (released March 8, 2019) (Fisher’s Exact test, Bonferroni correction) with the GO database released January 1, 2019, and annotation data set GO Biological Processes Complete (49, 107). Analysis of associated networks and upstream regulators was performed using IPA (http://www.qiagenbioinformatics.com/products/ingenuity-pathway-analysis) (53). 5′ and 3′ Splice site strengths were quantified using the webserver of MaxEntScan::score5ss and MaxEntScan::score3ss, respectively (http://hollywood.mit.edu/burgelab/maxent/) (108). Motif analysis was performed using the discriminative mode of the MEME tool in the MEME software package via the webserver (http://meme-suite.org/tools/meme) (68–70). BPS analysis was performed using the webserver of the SVM-BPfinder tool (http://regulatorygenomics.upf.edu/Software/SVM_BP) (79).

### Statistical analysis

Graph drawing and statistical analyses were performed using the GraphPad Prism software (version 8). Asterisks indicate statistical significance: ^*^P < 0.05, ^**^P < 0.01, ^***^P < 0.001, ^****^P < 0.0001. Unpaired *t* tests were performed for comparisons of two means. Kolmogorov–Smirnov normality tests were used to confirm that data were normally distributed (*P* < 0.05). Multiple testing adjustment was performed by two-way analysis of variance using the Bonferroni correction method.

### Data availability

The datasets used and/or analysed during the current study are available from the corresponding author on reasonable request. RNA-Seq datasets have been deposited with the Gene Expression Omnibus (GEO) under accession number GSE129619.

## Conflict of Interest statement

None declared.

## Funding

Medical Research Council (1916606; 1926882 to C.F.R. as part of a CASE studentship with Qiagen), Biotechnology and Biological Sciences Research Council (BB/N000358/1), National Institute for Health Research Manchester Biomedical Research Centre (IS-BRC-1215-20007 to W.G.N. and T.A.B.).

## Supplementary Material

SUPPLEMENTARY_MATERIAL_revised_ddz169

## References

[1] Will C.L. and Luhrmann R. (2011) Spliceosome structure and function. Cold Spring Harb. Perspect. Biol., 3, a003707. 21441581 10.1101/cshperspect.a003707 PMC3119917

[2] Matera A.G. and Wang Z. (2014) A day in the life of the spliceosome. Nat. Rev. Mol. Cell Biol., 15, 108–121. 24452469 10.1038/nrm3742 PMC4060434

[3] Jurica M.S. and Moore M.J. (2003) Pre-mRNA splicing. Mol. Cell, 12, 5–14. 12887888 10.1016/s1097-2765(03)00270-3

[4] Turunen J.J., Niemelä E.H., Verma B. and Frilander M.J. (2013) The significant other: splicing by the minor spliceosome. Wiley Interdiscip. Rev. RNA, 4, 61–76. 23074130 10.1002/wrna.1141 PMC3584512

[5] Wang Z. and Burge C.B. (2008) Splicing regulation: from a parts list of regulatory elements to an integrated splicing code. RNA, 14, 802–813. 18369186 10.1261/rna.876308 PMC2327353

[6] Scotti M.M. and Swanson M.S. (2016) RNA mis-splicing in disease. Nat. Rev. Genet., 17, 19–32. 26593421 10.1038/nrg.2015.3 PMC5993438

[7] Singh R.K. and Cooper T.A. (2012) Pre-mRNA splicing in disease and therapeutics. Trends Mol. Med., 18, 472–482. 22819011 10.1016/j.molmed.2012.06.006 PMC3411911

[8] Ward A.J. and Cooper T.A. (2010) The pathobiology of splicing. J. Pathol., 220, 152–163. 19918805 10.1002/path.2649 PMC2855871

[9] Růžičková Š. and Staněk D. (2017) Mutations in spliceosomal proteins and retina degeneration. RNA Biol., 14, 544–552. 27302685 10.1080/15476286.2016.1191735 PMC5449078

[10] Liu M.M. and Zack D.J. (2013) Alternative splicing and retinal degeneration. Clin. Genet., 84, 142–149. 23647439 10.1111/cge.12181 PMC4147722

[11] Morders D., Luo X., Kar A., Kuo D., Xu L., Fushimi K., YU G., Sternberg P. Jr. and Wu J.Y. (2009) Pre-mRNA splicing and retinitis pigmentosa. Mol. Vis., 12, 1259–1271. PMC2683577 17110909

[12] Pasternack S.M., Refke M., Paknia E., Hennies H.C., Franz T., Schäfer N., Fryer A., van Steensel M., Sweeney E., Just M. et al. (2013) Mutations in SNRPE, which encodes a core protein of the spliceosome, cause autosomal-dominant hypotrichosis simplex. Am. J. Hum. Genet., 92, 81–87. 23246290 10.1016/j.ajhg.2012.10.022 PMC3542472

[13] Lehalle D., Wieczorek D., Zechi-Ceide R.M., Passos-Bueno M.R., Lyonnet S., Amiel J. and Gordon C.T. (2015) A review of craniofacial disorders caused by spliceosomal defects. Clin. Genet., 88, 405–415. 25865758 10.1111/cge.12596

[14] Wieczorek D., Newman W.G., Wieland T., Berulava T., Kaffe M., Falkenstein D., Beetz C., Graf E., Schwarzmayr T., Douzgou S. et al. (2014) Compound heterozygosity of low-frequency promoter deletions and rare loss-of-function mutations in TXNL4A causes burn-McKeown syndrome. Am. J. Hum. Genet., 95, 698–707. 25434003 10.1016/j.ajhg.2014.10.014 PMC4259969

[15] Lines M.A., Huang L., Schwartzentruber J., Douglas S.L., Lynch D.C., Beaulieu C., Guion-Almeida M.L., Zechi-Ceide R.M., Gener B., Gillessen-Kaesbach G. et al. (2012) Haploinsufficiency of a spliceosomal GTPase encoded by EFTUD2 causes mandibulofacial dysostosis with microcephaly. Am. J. Hum. Genet., 90, 369–377. 22305528 10.1016/j.ajhg.2011.12.023 PMC3276671

[16] Xu M., Xie Y., Abouzeid H., Gordon C.T., Fiorentino A., Sun Z., Lehman A., Osman I.S., Dharmat R., Riveiro-Alvarez R. et al. (2017) Mutations in the Spliceosome component CWC27 cause retinal degeneration with or without additional developmental anomalies. Am. J. Hum. Genet., 100, 592–604. 28285769 10.1016/j.ajhg.2017.02.008 PMC5384039

[17] Wieczorek D. (2013) Human facial dysostoses. Clin. Genet., 83, 499–510. 23565775 10.1111/cge.12123

[18] Yu K.P.T., Luk H.-M., Gordon C.T., Fung G., Oufadem M., Garcia-Barcelo M.M., Amiel J., Chung B.H.Y., Lo I.F.M. and Tiong Y.T. (2018) Mandibulofacial dysostosis Guion-Almeida type caused by novel EFTUD2 splice site variants in two Asian children. Clin. Dysmorphol., 27, 31–35. 29381487 10.1097/MCD.0000000000000214

[19] Sarkar A., Emrick L.T., Smith E.M., Austin E.G., Yang Y., Hunter J.V., Scaglia F. and Lalani S.R. (2015) Novel de novo mutations in EFTUD2 detected by exome sequencing in mandibulofacial dysostosis with microcephaly syndrome. Am. J. Med. Genet. A, 167, 914–918. 10.1002/ajmg.a.36948 25735261

[20] Lehalle D., Gordon C.T., Oufadem M., Goudefroye G., Boutaud L., Alessandri J.-L., Baena N., Baujat G., Baumann C., Boute-Benejean O. et al. (2014) Delineation of EFTUD2 haploinsufficiency-related phenotypes through a series of 36 patients. Hum. Mutat., 35, 478–485. 24470203 10.1002/humu.22517

[21] Fabrizio P., Laggerbauer B., Lauber J., Lane W.S. and Lührmann R. (1997) An evolutionarily conserved U5 sn RNP-specific protein is a GTP-binding factor closely related to the ribosomal translocase EF-2. EMBO J., 16, 4092–4106. 9233818 10.1093/emboj/16.13.4092 PMC1170032

[22] Frazer L.N., Nancollis V. and O’Keefe R.T. (2008) The role of Snu 114p during pre-mRNA splicing: figure 1. Biochem. Soc. Trans., 36, 551–553. 18482006 10.1042/BST0360551

[23] Nguyen T.H.D., Galej W.P., Bai X., Oubridge C., Newman A.J., Scheres S.H.W. and Nagai K. (2016) Cryo-EM structure of the yeast U4/U6.U5 tri-sn RNP at 3.7 Å resolution. Nature, 530, 298–302. 26829225 10.1038/nature16940 PMC4762201

[24] Nguyen T.H.D., Galej W.P., Bai X., Savva C.G., Newman A.J., Scheres S.H.W. and Nagai K. (2015) The architecture of the spliceosomal U4/U6.U5 tri-sn RNP. Nature, 523, 47–52. 26106855 10.1038/nature14548 PMC4536768

[25] Rigo N., Sun C., Fabrizio P., Kastner B. and Luhrmann R. (2015) Protein localisation by electron microscopy reveals the architecture of the yeast spliceosomal B complex. EMBO J., 34, 3059–3073. 26582754 10.15252/embj.201592022 PMC4687791

[26] Nancollis V., Ruckshanthi J.P.D., Frazer L.N. and O’Keefe R.T. (2013) The U5 sn RNA internal loop 1 is a platform for Brr 2, Snu 114 and Prp 8 protein binding during U5 sn RNP assembly. J. Cell. Biochem., 114, 2770–2784. 23857713 10.1002/jcb.24625 PMC4065371

[27] Liu S. (2006) The network of protein-protein interactions within the human U4/U6.U5 tri-sn RNP. RNA, 12, 1418–1430. 16723661 10.1261/rna.55406 PMC1484429

[28] Agafonov D.E., Kastner B., Dybkov O., Hofele R.V., Liu W.-T., Urlaub H., Luhrmann R. and Stark H. (2016) Molecular architecture of the human U4/U6.U5 tri-sn RNP. Science, 351, 1416–1420. 26912367 10.1126/science.aad2085

[29] Bartels C. (2002) The ribosomal translocase homologue Snu 114p is involved in unwinding U4/U6 RNA during activation of the spliceosome. EMBO Rep., 3, 875–880. 12189173 10.1093/embo-reports/kvf172 PMC1084225

[30] Small E.C., Leggett S.R., Winans A.A. and Staley J.P. (2006) The EF-G-like GTPase Snu114p regulates spliceosome dynamics mediated by Brr2p, a DExD/H box ATPase. Mol. Cell, 23, 389–399. 16885028 10.1016/j.molcel.2006.05.043 PMC3777414

[31] Bartels C., Urlaub H., Lührmann R. and Fabrizio P. (2003) Mutagenesis suggests several roles of Snu114p in pre-mRNA splicing. J. Biol. Chem., 278, 28324–28334. 12736260 10.1074/jbc.M303043200

[32] Deml B., Reis L.M., Muheisen S., Bick D. and Semina E.V. (2015) EFTUD2 deficiency in vertebrates: identification of a novel human mutation and generation of a zebrafish model. Birth Defects Res. A Clin. Mol. Teratol., 103, 630–640. 26118977 10.1002/bdra.23397 PMC4487781

[33] Lei L., Yan S.-Y., Yang R., Chen J.-Y., Li Y., Bu Y., Chang N., Zhou Q., Zhu X., Li C.-Y. et al. (2017) Spliceosomal protein eftud2 mutation leads to p53-dependent apoptosis in zebrafish neural progenitors. Nucleic Acids Res., 45, 3422–3436. 27899647 10.1093/nar/gkw1043 PMC5389467

[34] Cong L., Ran F.A., Cox D., Lin S., Barretto R., Habib N., Hsu P.D., Wu X., Jiang W., Marraffini L.A. et al. (2013) Multiplex genome engineering using CRISPR/Cas systems. Science, 339, 819–823. 23287718 10.1126/science.1231143 PMC3795411

[35] Mali P., Aach J., Stranges P.B., Esvelt K.M., Moosburner M., Kosuri S., Yang L. and Church G.M. (2013) CAS9 transcriptional activators for target specificity screening and paired nickases for cooperative genome engineering. Nat. Biotechnol., 31, 833–838. 23907171 10.1038/nbt.2675 PMC3818127

[36] Cho S.W., Kim S., Kim Y., Kweon J., Kim H.S., Bae S. and Kim J.-S. (2014) Analysis of off-target effects of CRISPR/Cas-derived RNA-guided endonucleases and nickases. Genome Res., 24, 132–141. 24253446 10.1101/gr.162339.113 PMC3875854

[37] Ran F.A., Hsu P.D., Lin C.-Y., Gootenberg J.S., Konermann S., Trevino A.E., Scott D.A., Inoue A., Matoba S., Zhang Y. et al. (2013) Double nicking by RNA-guided CRISPR Cas9 for enhanced genome editing specificity. Cell, 154, 1380–1389. 23992846 10.1016/j.cell.2013.08.021 PMC3856256

[38] Yang L., Yang J.L., Byrne S., Pan J. and Church G.M. (2014) CRISPR/Cas9-directed genome editing of cultured cells In Current Protocols in Molecular Biology. John Wiley & Sons, Inc., Hoboken, NJ, USA, pp. 31.1.1–31.1.17. 10.1002/0471142727.mb3101s107 24984853

[39] Togashi Y., Mizuuchi H., Tomida S., Terashima M., Hayashi H., Nishio K. and Mitsudomi T. (2015) MET gene exon 14 deletion created using the CRISPR/Cas9 system enhances cellular growth and sensitivity to a MET inhibitor. Lung Cancer, 90, 590–597. 26547802 10.1016/j.lungcan.2015.10.020

[40] Malankhanova T.B., Malakhova A.A., Medvedev S.P. and Zakian S.M. (2017) Modern genome editing technologies in Huntington’s disease research. J. Huntingtons Dis., 6, 19–31. 28128770 10.3233/JHD-160222 PMC5389024

[41] Martinez-Lage M., Torres-Ruiz R. and Rodriguez-Perales S. (2017) CRISPR/Cas9 technology: applications and human disease modeling. Prog. Mol. Biol. Transl. Sci., 152, 23–48. 29150003 10.1016/bs.pmbts.2017.09.002

[42] Sakaki K., Yoshina S., Shen X., Han J., DeSantis M.R., Xiong M., Mitani S. and Kaufman R.J. (2012) RNA surveillance is required for endoplasmic reticulum homeostasis. Proc. Natl. Acad. Sci., 109, 8079–8084. 22562797 10.1073/pnas.1110589109 PMC3361423

[43] Ron D. and Walter P. (2007) Signal integration in the endoplasmic reticulum unfolded protein response. Nat. Rev. Mol. Cell Biol., 8, 519–529. 17565364 10.1038/nrm2199

[44] Gardner B.M., Pincus D., Gotthardt K., Gallagher C.M. and Walter P. (2013) Endoplasmic reticulum stress sensing in the unfolded protein response. Cold Spring Harb. Perspect. Biol., 5, a013169. 23388626 10.1101/cshperspect.a013169 PMC3578356

[45] Tabara K., Iwata Y. and Koizumi N. (2018) The unfolded protein response. Methods Mol. Biol., 1691, 223–230. 29043681 10.1007/978-1-4939-7389-7_17

[46] Sano R. and Reed J.C. (2013) ER stress-induced cell death mechanisms. Biochim. Biophys. Acta, 1833, 3460–3470. 23850759 10.1016/j.bbamcr.2013.06.028 PMC3834229

[47] Joshi A., Newbatt Y., McAndrew P.C., Stubbs M., Burke R., Richards M.W., Bhatia C., Caldwell J.J., McHardy T., Collins I. et al. (2015) Molecular mechanisms of human IRE1 activation through dimerization and ligand binding. Oncotarget, 6, 13019–13035. 25968568 10.18632/oncotarget.3864 PMC4536996

[48] Yoshida H., Matsui T., Yamamoto A., Okada T. and Mori K. (2001) XBP1 mRNA is induced by ATF6 and spliced by IRE1 in response to ER stress to produce a highly active transcription factor. Cell, 107, 881–891. 11779464 10.1016/s0092-8674(01)00611-0

[49] Mi H., Huang X., Muruganujan A., Tang H., Mills C., Kang D. and Thomas P.D. (2017) PANTHER version 11: expanded annotation data from gene ontology and Reactome pathways, and data analysis tool enhancements. Nucleic Acids Res., 45, D183–D189. 27899595 10.1093/nar/gkw1138 PMC5210595

[50] Wu G., Fan L., Edmonson M.N., Shaw T., Boggs K., Easton J., Rusch M.C., Webb T.R., Zhang J. and Potter P.M. (2018) Inhibition of SF3B1 by molecules targeting the spliceosome results in massive aberrant exon skipping. RNA, 24, 1056–1066. 29844105 10.1261/rna.065383.117 PMC6049506

[51] Trede N.S., Medenbach J., Damianov A., Hung L.-H., Weber G.J., Paw B.H., Zhou Y., Hersey C., Zapata A., Keefe M. et al. (2007) Network of coregulated spliceosome components revealed by zebrafish mutant in recycling factor p 110. Proc. Natl. Acad. Sci., 104, 6608–6613. 17416673 10.1073/pnas.0701919104 PMC1871833

[52] Schreib C.C., Bowman E.K., Hernandez C.A., Lucas A.L., Potts C.H.S. and Maeder C. (2018) Functional and biochemical characterization of Dib1’s role in pre-messenger RNA splicing. J. Mol. Biol., 430, 1640–1651. 29715471 10.1016/j.jmb.2018.04.027 PMC6155989

[53] Krämer A., Green J., Pollard J. and Tugendreich S. (2014) Causal analysis approaches in Ingenuity Pathway Analysis. Bioinformatics, 30, 523–530. 24336805 10.1093/bioinformatics/btt703 PMC3928520

[54] Iwata J., Suzuki A., Pelikan R.C., Ho T.-V. and Chai Y. (2013) Noncanonical transforming growth factor β (TGFβ) Signaling in cranial neural crest cells causes tongue muscle developmental defects. J. Biol. Chem., 288, 29760–29770. 23950180 10.1074/jbc.M113.493551 PMC3795273

[55] Iwata J., Parada C. and Chai Y. (2011) The mechanism of TGF-β signaling during palate development. Oral Dis., 17, 733–744. 21395922 10.1111/j.1601-0825.2011.01806.x PMC3329177

[56] Dudas M. and Kaartinen V. (2005) TGF-β superfamily and mouse craniofacial development: interplay of morphogenetic proteins and receptor signaling controls normal formation of the face. Curr. Top. Dev. Biol., 66, 65–133. 15797452 10.1016/S0070-2153(05)66003-6

[57] Pezzini A., Del Zotto E., Giossi A., Volonghi I., Costa P. and Padovani A. (2012) Transforming growth factor β signaling perturbation in the Loeys–Dietz syndrome. Curr. Med. Chem., 19, 454–460. 22335518 10.2174/092986712803414286

[58] Shen S., Park J.W., Lu Z., Lin L., Henry M.D., Wu Y.N., Zhou Q. and Xing Y. (2014) rMATS: robust and flexible detection of differential alternative splicing from replicate RNA-Seq data. Proc. Natl. Acad. Sci., 111, E5593–E5601. 25480548 10.1073/pnas.1419161111 PMC4280593

[59] Anders S., Reyes A. and Huber W. (2012) Detecting differential usage of exons from RNA-seq data. Genome Res., 22, 2008–2017. 22722343 10.1101/gr.133744.111 PMC3460195

[60] Shen S., Park J.W., Huang J., Dittmar K.A., Lu Z., Zhou Q., Carstens R.P. and Xing Y. (2012) MATS: a Bayesian framework for flexible detection of differential alternative splicing from RNA-Seq data. Nucleic Acids Res., 40, e61–e61. 22266656 10.1093/nar/gkr1291 PMC3333886

[61] Marques F., Tenney J., Duran I., Martin J., Nevarez L., Pogue R., Krakow D., Cohn D.H. and Li B. (2016) Altered mRNA splicing, chondrocyte gene expression and abnormal skeletal development due to SF3B4 mutations in Rodriguez acrofacial dysostosis. PLoS Genet., 12, e1006307. 27622494 10.1371/journal.pgen.1006307 PMC5021280

[62] Park E., Pan Z., Zhang Z., Lin L. and Xing Y. (2018) The expanding landscape of alternative splicing variation in human populations. Am. J. Hum. Genet., 102, 11–26. 29304370 10.1016/j.ajhg.2017.11.002 PMC5777382

[63] Merkuri F. and Fish J.L. (2019) Developmental processes regulate craniofacial variation in disease and evolution. Genesis, 57, e23249. 30207415 10.1002/dvg.23249 PMC6349473

[64] Calo E., Gu B., Bowen M.E., Aryan F., Zalc A., Liang J., Flynn R.A., Swigut T., Chang H.Y., Attardi L.D. et al. (2018) Tissue-selective effects of nucleolar stress and rDNA damage in developmental disorders. Nature, 554, 112–117. 29364875 10.1038/nature25449 PMC5927778

[65] Rinon A., Molchadsky A., Nathan E., Yovel G., Rotter V., Sarig R. and Tzahor E. (2011) p53 coordinates cranial neural crest cell growth and epithelial-mesenchymal transition/delamination processes. Development, 138, 1827–1838. 21447558 10.1242/dev.053645

[66] Dixon J. (2000) Increased levels of apoptosis in the prefusion neural folds underlie the craniofacial disorder, Treacher Collins syndrome. Hum. Mol. Genet., 9, 1473–1480. 10888597 10.1093/hmg/9.10.1473

[67] Obulesu M. and Lakshmi M.J. (2014) Apoptosis in Alzheimer’s disease: an understanding of the physiology, pathology and therapeutic avenues. Neurochem. Res., 39, 2301–2312. 25322820 10.1007/s11064-014-1454-4

[68] Bailey T.L., Johnson J., Grant C.E. and Noble W.S. (2015) The MEME suite. Nucleic Acids Res., 43, W39–W49. 25953851 10.1093/nar/gkv416 PMC4489269

[69] Bailey T.L., Boden M., Buske F.A., Frith M., Grant C.E., Clementi L., Ren J., Li W.W. and Noble W.S. (2009) MEME SUITE: tools for motif discovery and searching. Nucleic Acids Res., 37, W202–W208. 19458158 10.1093/nar/gkp335 PMC2703892

[70] Bailey T.L., Williams N., Misleh C. and Li W.W. (2006) MEME: discovering and analyzing DNA and protein sequence motifs. Nucleic Acids Res., 34, W369–W373. 16845028 10.1093/nar/gkl198 PMC1538909

[71] Holbrook J.A., Neu-Yilik G., Gehring N.H., Kulozik A.E. and Hentze M.W. (2006) Internal ribosome entry sequence-mediated translation initiation triggers nonsense-mediated decay. EMBO Rep., 7, 722–726. 16799467 10.1038/sj.embor.7400721 PMC1500827

[72] Lykke-Andersen S. and Jensen T.H. (2015) Nonsense-mediated mRNA decay: an intricate machinery that shapes transcriptomes. Nat. Rev. Mol. Cell Biol., 16, 665–677. 26397022 10.1038/nrm4063

[73] Gordon C.T., Petit F., Oufadem M., Decaestecker C., Jourdain A.-S., Andrieux J., Malan V., Alessandri J.-L., Baujat G., Baumann C. et al. (2012) EFTUD2 haploinsufficiency leads to syndromic oesophageal atresia. J. Med. Genet., 49, 737–746. 23188108 10.1136/jmedgenet-2012-101173

[74] Burow D.A., Umeh-Garcia M.C., True M.B., Bakhaj C.D., Ardell D.H. and Cleary M.D. (2015) Dynamic regulation of mRNA decay during neural development. Neural Dev., 10, 1–16. 25896902 10.1186/s13064-015-0038-6 PMC4413985

[75] Gurok U. (2004) Gene expression changes in the course of neural progenitor cell differentiation. J. Neurosci., 24, 5982–6002. 15229246 10.1523/JNEUROSCI.0809-04.2004 PMC6729244

[76] Su C.-H., Dhananjaya D. and Tarn W.-Y. (2018) Alternative splicing in neurogenesis and brain development. Front. Mol. Biosci., 5, 1–9. 29484299 10.3389/fmolb.2018.00012 PMC5816070

[77] Weyn-Vanhentenryck S.M., Feng H., Ustianenko D., Duffié R., Yan Q., Jacko M., Martinez J.C., Goodwin M., Zhang X., Hengst U. et al. (2018) Precise temporal regulation of alternative splicing during neural development. Nat. Commun., 9, 2189. 29875359 10.1038/s41467-018-04559-0 PMC5989265

[78] Middleton R., Gao D., Thomas A., Singh B., Au A., Wong J.J.L., Bomane A., Cosson B., Eyras E., Rasko J.E.J. et al. (2017) IRFinder: assessing the impact of intron retention on mammalian gene expression. Genome Biol., 18, 51. 28298237 10.1186/s13059-017-1184-4 PMC5353968

[79] Corvelo A., Hallegger M., Smith C.W.J. and Eyras E. (2010) Genome-wide association between branch point properties and alternative splicing. PLoS Comput. Biol., 6, e1001016. 10.1371/journal.pcbi.1001016 PMC2991248 21124863

[80] Kandul N.P. and Noor M.A.F. (2009) Large introns in relation to alternative splicing and gene evolution: a case study of drosophila Bruno-3. BMC Genet., 10, 67. 19840385 10.1186/1471-2156-10-67 PMC2767349

[81] Miriami E. (2003) Conserved sequence elements associated with exon skipping. Nucleic Acids Res., 31, 1974–1983. 12655015 10.1093/nar/gkg279 PMC152795

[82] Reja R., Vinayachandran V., Ghosh S. and Pugh B.F. (2015) Molecular mechanisms of ribosomal protein gene coregulation. Genes Dev., 29, 1942–1954. 26385964 10.1101/gad.268896.115 PMC4579351

[83] Braunschweig U., Barbosa-Morais N.L., Pan Q., Nachman E.N., Alipanahi B., Gonatopoulos-Pournatzis T., Frey B., Irimia M. and Blencowe B.J. (2014) Widespread intron retention in mammals functionally tunes transcriptomes. Genome Res., 24, 1774–1786. 25258385 10.1101/gr.177790.114 PMC4216919

[84] Sakabe N. and de Souza S. (2007) Sequence features responsible for intron retention in human. BMC Genomics, 8, 59. 17324281 10.1186/1471-2164-8-59 PMC1831480

[85] Meyer M., Plass M., Pérez-Valle J., Eyras E. and Vilardell J. (2011) Deciphering 3′ss selection in the yeast genome reveals an RNA Thermosensor that mediates alternative splicing. Mol. Cell, 43, 1033–1039. 21925391 10.1016/j.molcel.2011.07.030

[86] Payer L.M., Steranka J.P., Ardeljan D., Walker J., Fitzgerald K.C., Calabresi P.A., Cooper T.A. and Burns K.H. (2019) Alu insertion variants alter mRNA splicing. Nucleic Acids Res., 47, 421–431. 30418605 10.1093/nar/gky1086 PMC6326789

[87] Fredericks A., Cygan K., Brown B. and Fairbrother W. (2015) RNA-binding proteins: splicing factors and disease. Biomolecules, 5, 893–909. 25985083 10.3390/biom5020893 PMC4496701

[88] Chen W., Feng P., Ding H. and Lin H. (2018) Classifying included and excluded exons in exon skipping event using histone modifications. Front. Genet., 9, 1–7. 30327665 10.3389/fgene.2018.00433 PMC6174203

[89] Mao H., McMahon J.J., Tsai Y.-H., Wang Z. and Silver D.L. (2016) Haploinsufficiency for Core exon junction complex components disrupts embryonic neurogenesis and causes p53-mediated microcephaly. PLoS Genet., 12, e1006282. 27618312 10.1371/journal.pgen.1006282 PMC5019403

[90] Mao H., Brown H.E. and Silver D.L. (2017) Mouse models of Casc 3 reveal developmental functions distinct from other components of the exon junction complex. RNA, 23, 23–31. 27780844 10.1261/rna.058826.116 PMC5159646

[91] Miller E.E., Kobayashi G.S., Musso C.M., Allen M., Ishiy F.A.A., de Caires L.C., Goulart E., Griesi-Oliveira K., Zechi-Ceide R.M., Richieri-Costa A. et al. (2017) EIF4A3 deficient human iPSCs and mouse models demonstrate neural crest defects that underlie Richieri-Costa-Pereira syndrome. Hum. Mol. Genet., 26, 2177–2191. 28334780 10.1093/hmg/ddx078 PMC5731442

[92] Devotta A., Juraver-Geslin H., Gonzalez J.A., Hong C.-S. and Saint-Jeannet J.-P. (2016) Sf3b4-depleted Xenopus embryos: a model to study the pathogenesis of craniofacial defects in Nager syndrome. Dev. Biol., 415, 371–382. 26874011 10.1016/j.ydbio.2016.02.010 PMC4914463

[93] Fabrizio P., Dannenberg J., Dube P., Kastner B., Stark H., Urlaub H. and Lührmann R. (2009) The evolutionarily conserved core design of the catalytic activation step of the yeast spliceosome. Mol. Cell, 36, 593–608. 19941820 10.1016/j.molcel.2009.09.040

[94] Zhang X., Yan C., Hang J., Finci L.I., Lei J. and Shi Y. (2017) An atomic structure of the human spliceosome. Cell, 169, 918–929. 28502770 10.1016/j.cell.2017.04.033

[95] Hegele A., Kamburov A., Grossmann A., Sourlis C., Wowro S., Weimann M., Will C.L., Pena V., Lührmann R. and Stelzl U. (2012) Dynamic protein-protein interaction wiring of the human spliceosome. Mol. Cell, 45, 567–580. 22365833 10.1016/j.molcel.2011.12.034

[96] Labun K., Montague T.G., Gagnon J.A., Thyme S.B. and Valen E. (2016) CHOPCHOP v2: a web tool for the next generation of CRISPR genome engineering. Nucleic Acids Res., 44, W272–W276. 27185894 10.1093/nar/gkw398 PMC4987937

[97] Montague T.G., Cruz J.M., Gagnon J.A., Church G.M. and Valen E. (2014) CHOPCHOP: a CRISPR/Cas9 and TALEN web tool for genome editing. Nucleic Acids Res., 42, W401–W407. 24861617 10.1093/nar/gku410 PMC4086086

[98] Frazer L.N., Lovell S.C. and O’Keefe R.T. (2009) Analysis of synthetic lethality reveals genetic interactions between the GTPase Snu114p and sn RNAs in the catalytic core of the *Saccharomyces cerevisiae* spliceosome. Genetics, 183, 497–515. 19620389 10.1534/genetics.109.107243 PMC2766312

[99] Wang X., Spandidos A., Wang H. and Seed B. (2012) PrimerBank: a PCR primer database for quantitative gene expression analysis, 2012 update. Nucleic Acids Res., 40, D1144–D1149. 22086960 10.1093/nar/gkr1013 PMC3245149

[100] Spandidos A., Wang X., Wang H. and Seed B. (2010) PrimerBank: a resource of human and mouse PCR primer pairs for gene expression detection and quantification. Nucleic Acids Res., 38, D792–D799. 19906719 10.1093/nar/gkp1005 PMC2808898

[101] Kozera B. and Rapacz M. (2013) Reference genes in real-time PCR. J. Appl. Genet., 54, 391–406. 24078518 10.1007/s13353-013-0173-x PMC3825189

[102] Ye J., Coulouris G., Zaretskaya I., Cutcutache I., Rozen S. and Madden T.L. (2012) Primer-BLAST: a tool to design target-specific primers for polymerase chain reaction. BMC Bioinformatics, 13, 134. 22708584 10.1186/1471-2105-13-134 PMC3412702

[103] Bolger A.M., Lohse M. and Usadel B. (2014) Trimmomatic: a flexible trimmer for Illumina sequence data. Bioinformatics, 30, 2114–2120. 24695404 10.1093/bioinformatics/btu170 PMC4103590

[104] Dobin A., Davis C.A., Schlesinger F., Drenkow J., Zaleski C., Jha S., Batut P., Chaisson M. and Gingeras T.R. (2013) STAR: ultrafast universal RNA-seq aligner. Bioinformatics, 29, 15–21. 23104886 10.1093/bioinformatics/bts635 PMC3530905

[105] Love M.I., Huber W. and Anders S. (2014) Moderated estimation of fold change and dispersion for RNA-seq data with DESeq2. Genome Biol., 15, 550. 25516281 10.1186/s13059-014-0550-8 PMC4302049

[106] Li B. and Dewey C.N. (2014) RSEM: accurate transcript quantification from RNA-Seq data with or without a reference genome. BMC Bioinformatics, 12, 323. 10.1186/1471-2105-12-323 PMC3163565 21816040

[107] Mi H., Muruganujan A., Casagrande J.T. and Thomas P.D. (2013) Large-scale gene function analysis with the PANTHER classification system. Nat. Protoc., 8, 1551–1566. 23868073 10.1038/nprot.2013.092 PMC6519453

[108] Yeo G. and Burge C.B. (2004) Maximum entropy Modeling of short sequence motifs with applications to RNA splicing signals. J. Comput. Biol., 11, 377–394. 15285897 10.1089/1066527041410418

