# Supplementary material for: Disease modeling of core pre-mRNA splicing factor haploinsufficiency
Source: Hum Mol Genet. 2019 Jul 13;28(22):3704–23. doi: 10.1093/hmg/ddz169 (PMC6935387; doi:10.1093/hmg/ddz169)
Supplement: SUPPLEMENTARY_MATERIAL_revised_ddz169 [file supplementary_material_revised_ddz169.pdf]

SUPPLEMENTARY MATERIAL

Disease modelling of core pre-mRNA splicing factor haploinsufficiency

Katherine A. Wood[1,2], Charles F. Rowlands[1,2], Wasay Mohiuddin Shaikh Qureshi[1], Huw B. Thomas[1], Weronika A. Buczek[1], Tracy A. Briggs[1,2], Simon J. Hubbard[1], Kathryn Hentges[1], William G. Newman[1,2], Raymond T. O'Keefe[1*]

*Corresponding author: rokeefe@manchester.ac.uk

THIS DOCUMENT CONTAINS:

SUPPLEMENTARY FIGURES 1 – 8
SUPPLEMENTARY TABLES 1 – 4
SUPPLEMENTARY PRIMER TABLES 1 – 4

SUPPLEMENTARY FIGURE 1

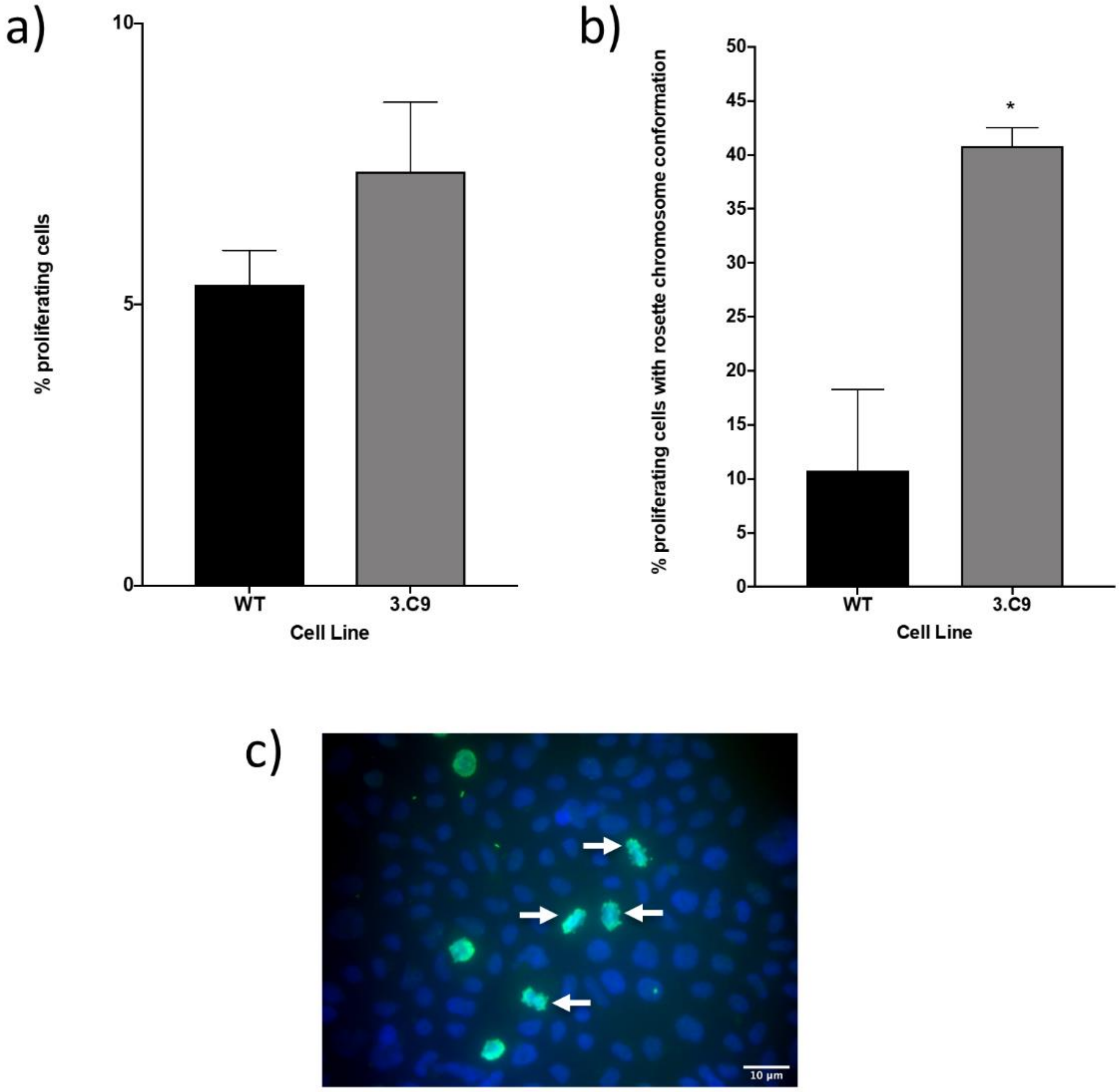

Supplementary Figure 1. Analysis of proliferating, mitotic, cells by detecting histone H3 serine 10 phosphorylation.

Both wildtype and 3.C9 cell lines were fixed and histone H3 serine 10 phosphorylation, indicative of chromosome condensation in mitotic cells, was detected by immunofluorescent labelling. **a)** percentage of cells positive for histone H3 serine 10 phosphorylation in wildtype and 3.C9 cells. **b)** percentage of histone H3 serine 10 phosphorylation positive cells with a rosette chromosome conformation, * p-value < 0.05. **c)** immunofluorescent labelling to detect histone H3 serine 10 phosphorylation in mitotic cells (green) in the 3.C9 cell line. Rosette like chromosome conformation is indicated with white arrows. DNA is labelled with DAPI (blue).

SUPPLEMENTARY FIGURE 2

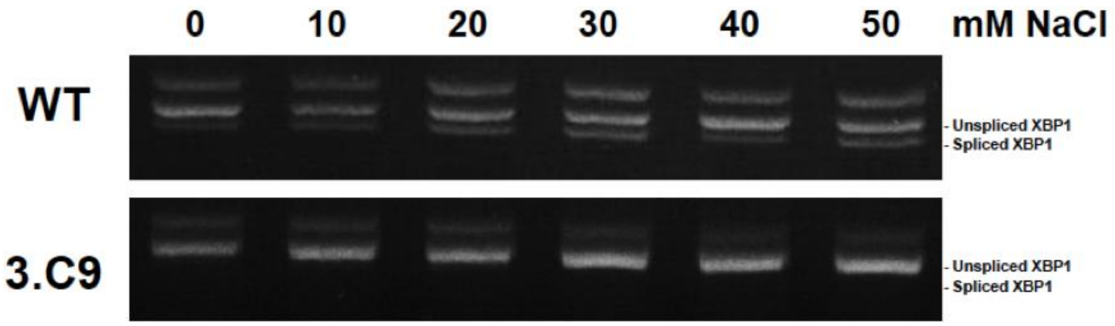

Supplementary Figure 2. *XBP1* splicing in WT and 3.C9 cells following NaCl treatment. *XBP1* splicing in WT and 3.C9 cells following five hours NaCl treatment at increasing concentrations. cDNA was made from cells lines following five hours growth at 37˚C in 5% CO_2 with and without NaCl, and was used as a template in RT-PCR reactions with primers which amplify both the spliced and unspliced forms of *XBP1*.

SUPPLEMENTARY FIGURE 3

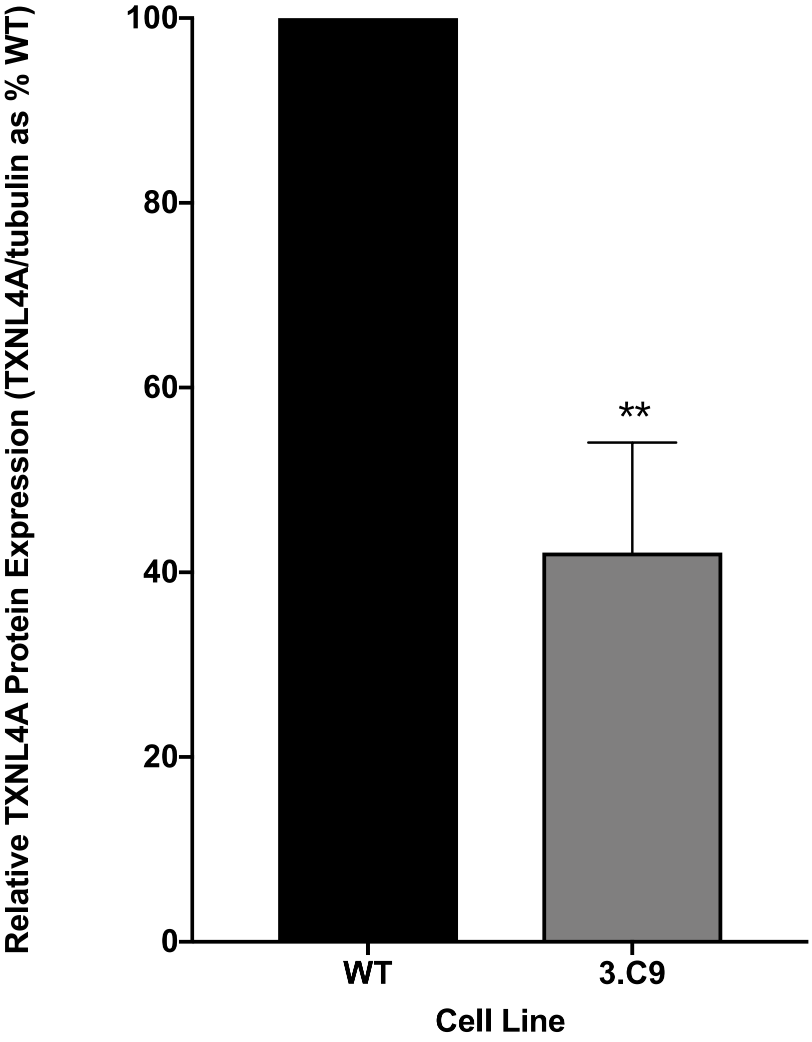

Supplementary Figure 3. Relative protein levels for TXNL4A in 3.C9 cells compared to WT cells.

Relative protein levels were determined using western blotting of total cell extracts. Signals were quantified using LI-COR Image Studio software, normalising the TXNL4A signal to α-tubulin, and values expressed as percentages of WT controls. Graphs show standard error of the mean of three experiments. ** p-value < 0.01.

SUPPLEMENTARY FIGURE 4

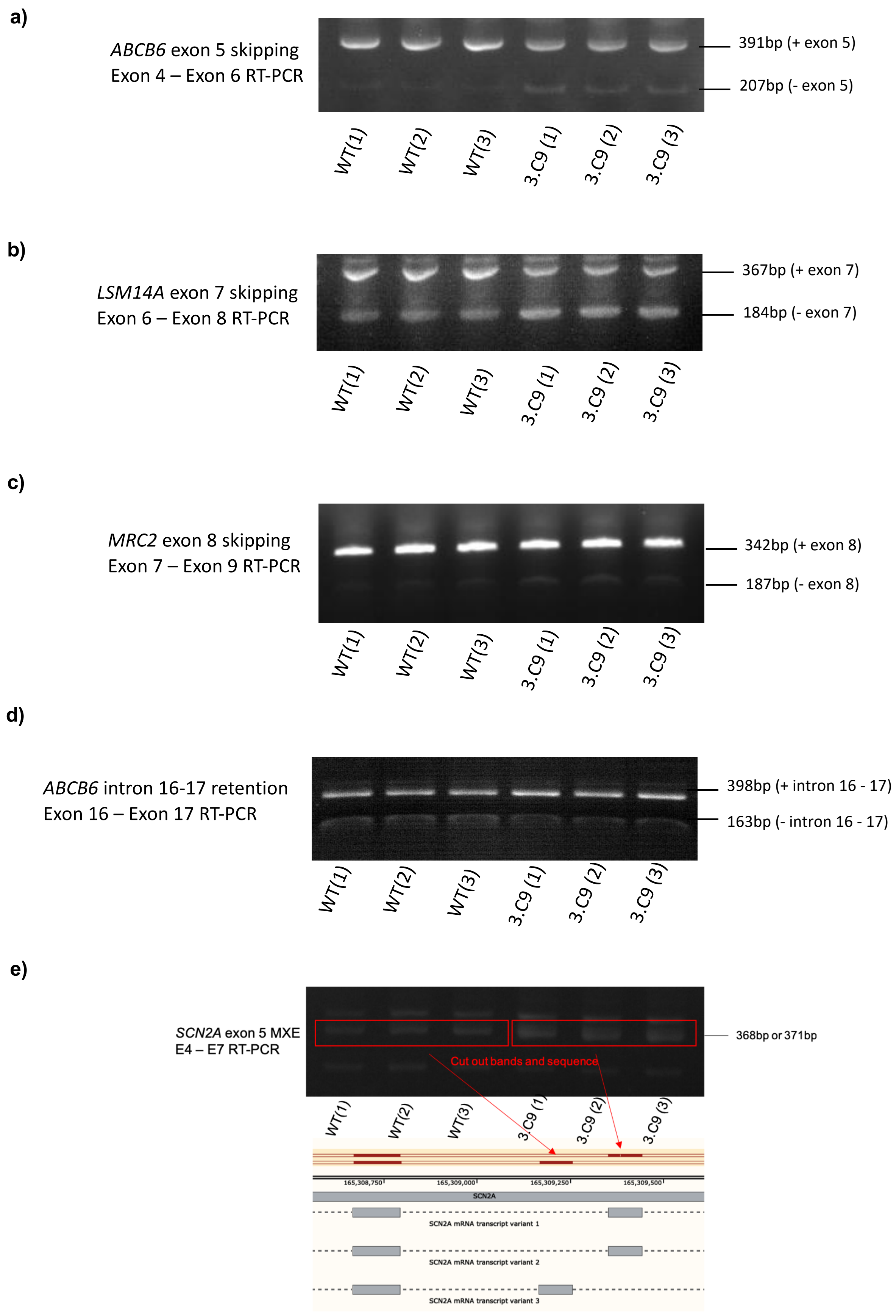

Supplementary Figure 4. Validation of RNA-Seq expression patterns using RT-PCR.
cDNA made from RNA generated in the same preparation as RNA used in RNA-Seq experiments was used as a template for RT-PCR reactions with primers amplifying **a)** *ABCB6* exon 4 to exon 6; **b)** *LSM14A* exon 6 to exon 8; **c)** *MRC2* exon 7 to exon 9; **d)** *ABCB6* exon 16 to exon 17; **e)** *SCN2A* exon 4 to exon 7. The *SCN2A* RT-PCR product was purified and sequenced to show mutually exclusive usage of two isoforms of exon 5. MXE = mutually exclusive exons. RT-PCRs were performed in triplicate using cDNA generated from three of the six RNA samples submitted for RNA-Seq for each cell line.

SUPPLEMENTARY FIGURE 5

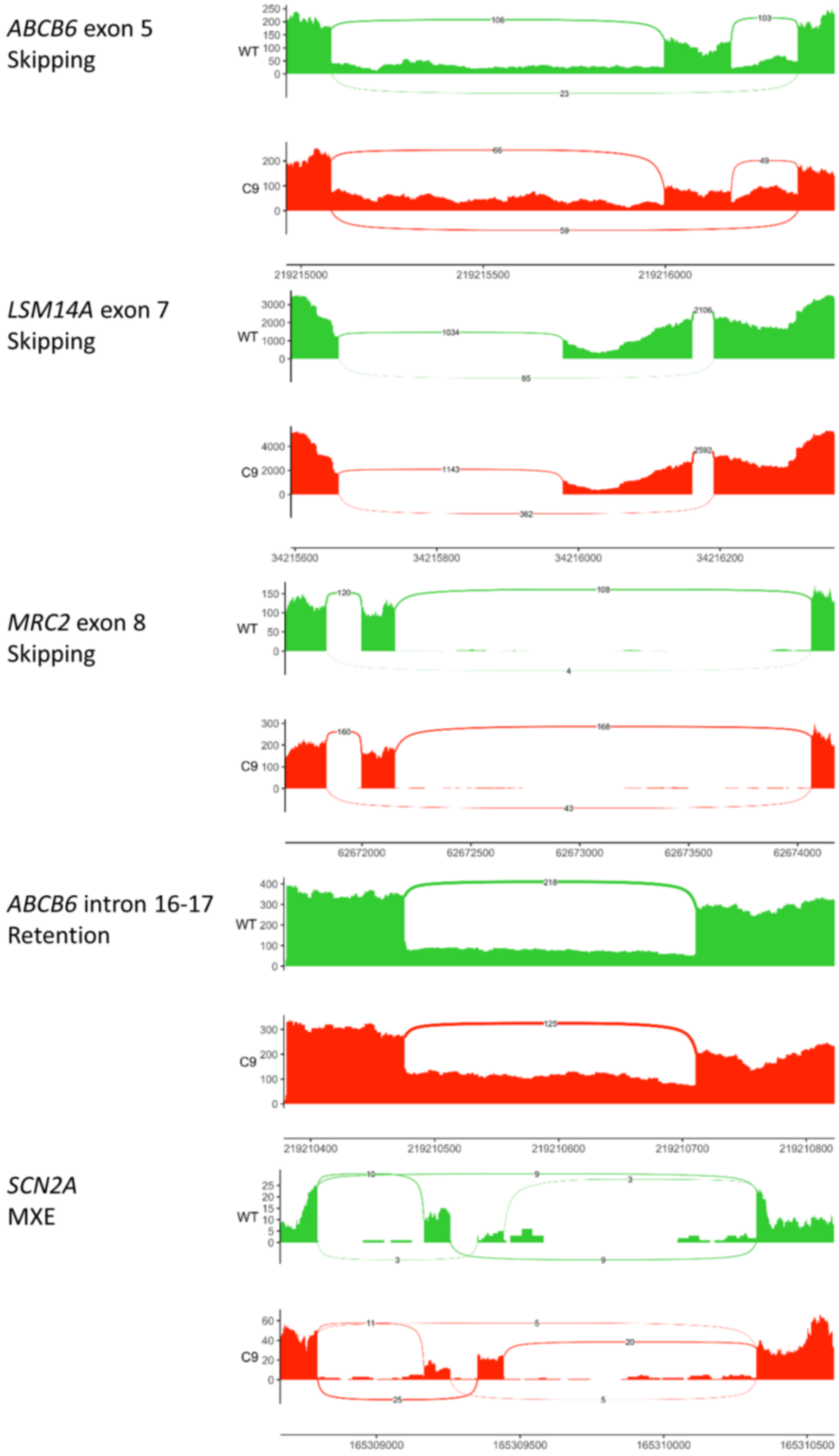

Supplementary Figure 5. Sashimi plots of RNA-seq data for examples validated by RT-PCR

SUPPLEMENTARY FIGURE 6

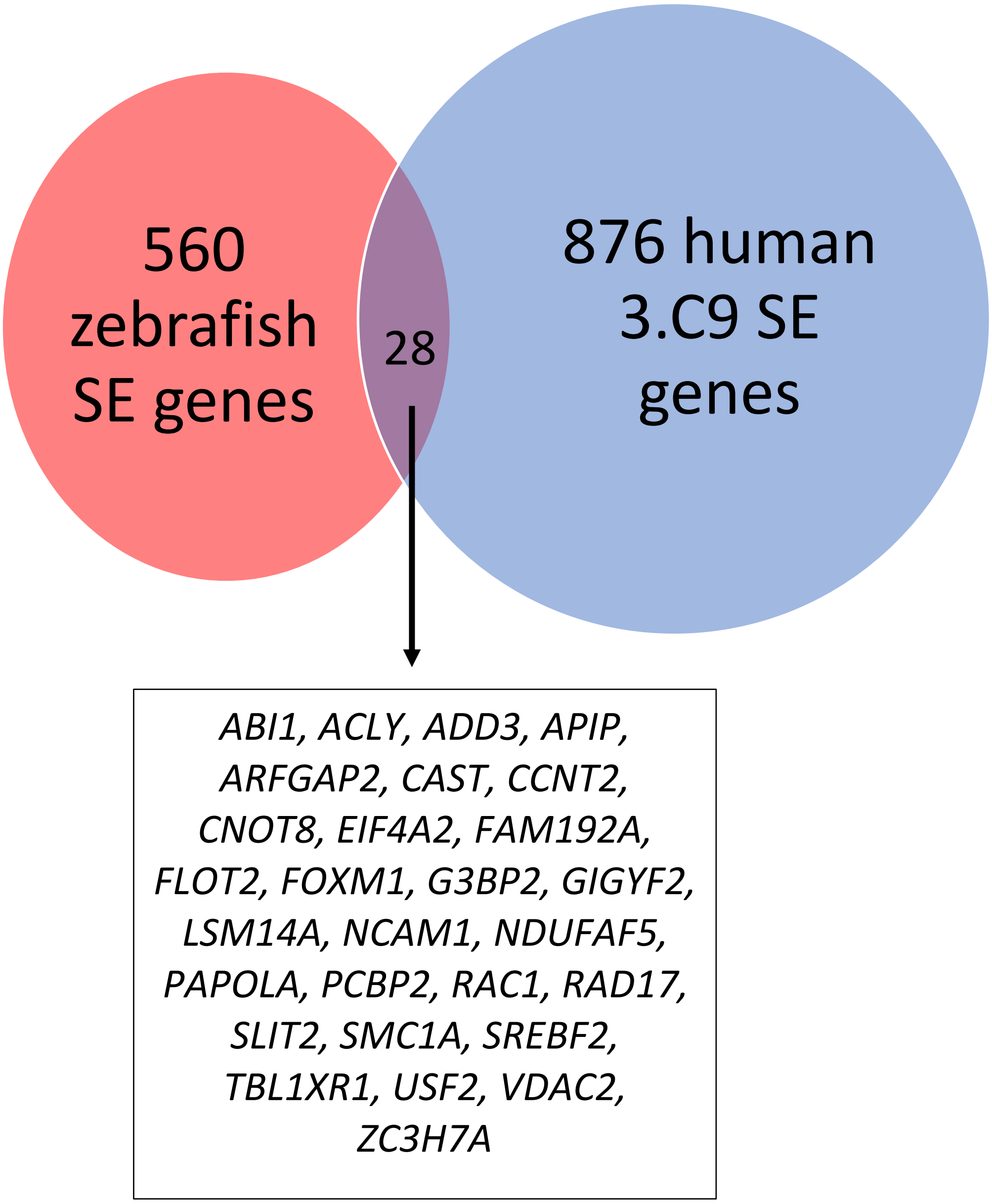

Supplementary Figure 6. Overlapping exon skipped genes in 3.C9 *EFTUD2*-knockdown human cell line and *eftud2*-knockdown zebrafish model (Lei et al., 2017).
Venn diagram showing the identity of the 28 genes which displayed altered exon skipping both in the *eftud2*-knockdown zebrafish model and the 3.C9 *EFTUD2*-knockdown human cell line. SE = skipped exon.

SUPPLEMENTARY FIGURE 7

a)

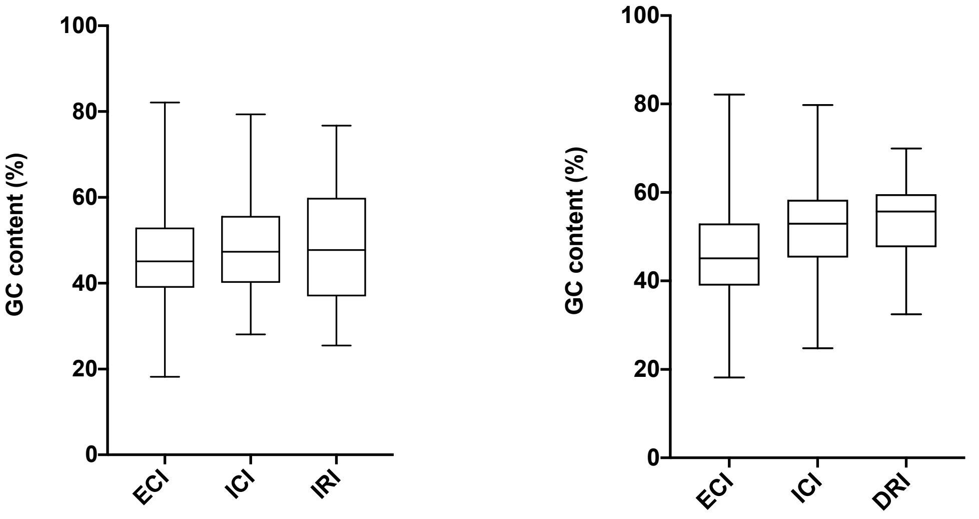

b)

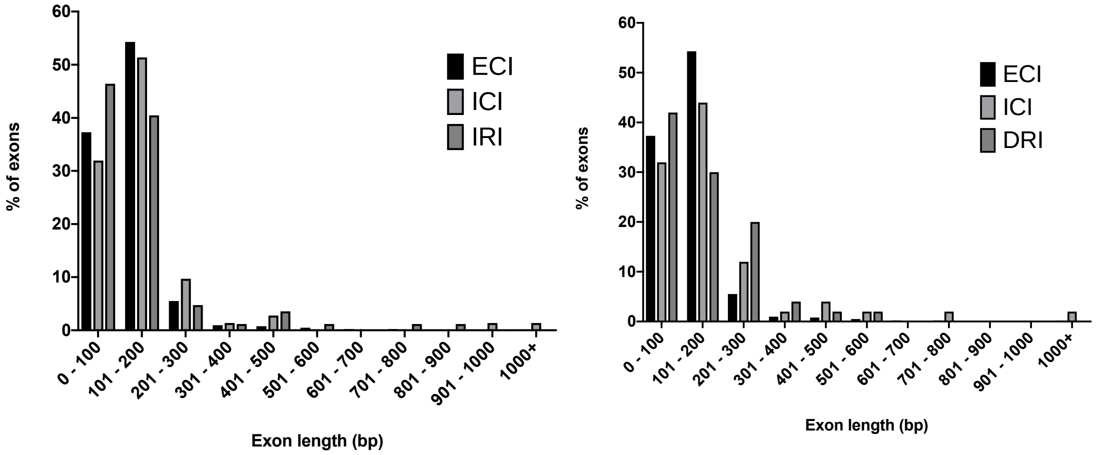

c)

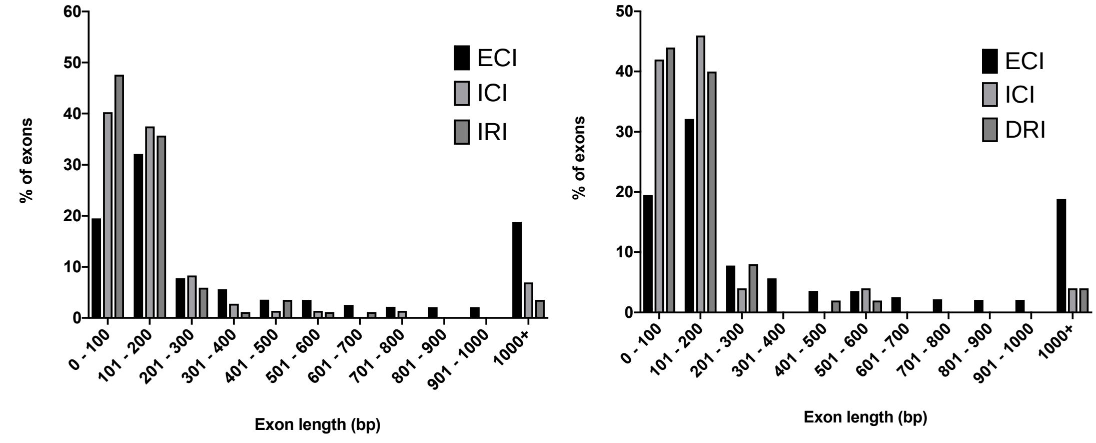

d)

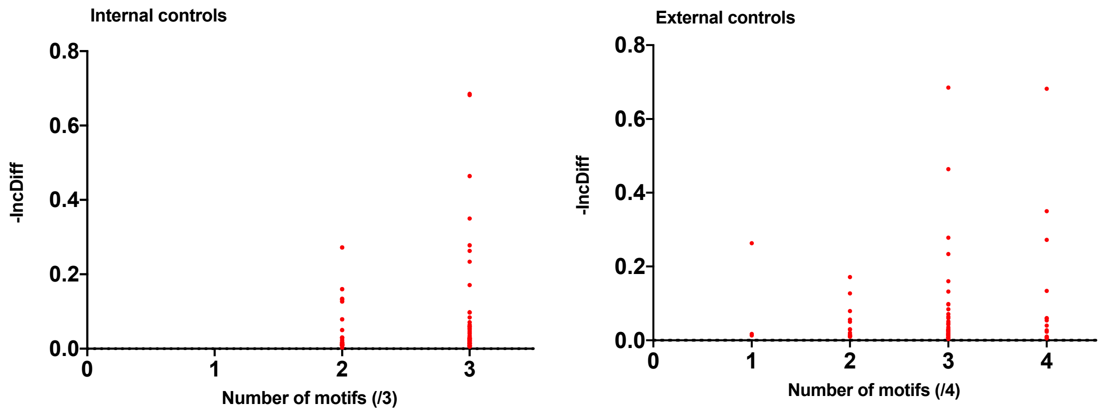

e)

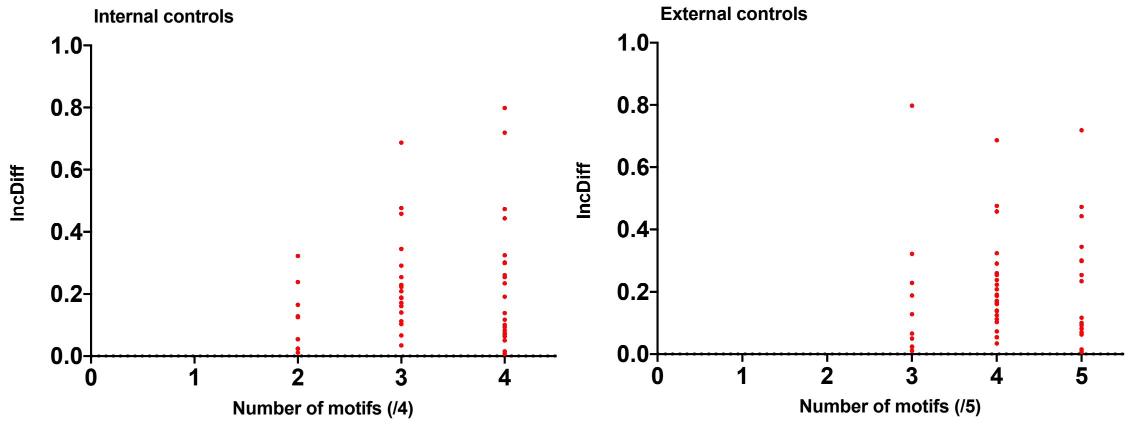

Supplementary Figure 7. Characteristics of retained introns.

a) GC content, **b)** upstream exon length and **c)** downstream exon length for introns showing increased retention (IRI) and introns showing decreased retention (DRI) in 3.C9 cells, compared to external control introns (ECI) and internal control introns (ICI). **d)** Correlation between number of enriched motifs in retained intron E-I-E sequences and severity of intron retention for introns displaying increased retention in 3.C9 cells. **e)** Correlation between number of enriched motifs in retained intron E-I-E sequences and severity of intron retention for introns showing decreased retention in 3.C9 cells.

SUPPLEMENTARY FIGURE 8

a)

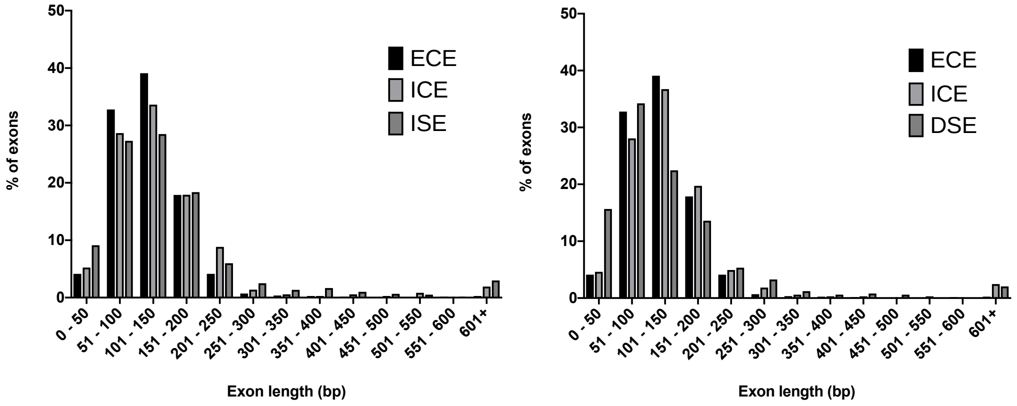

b)

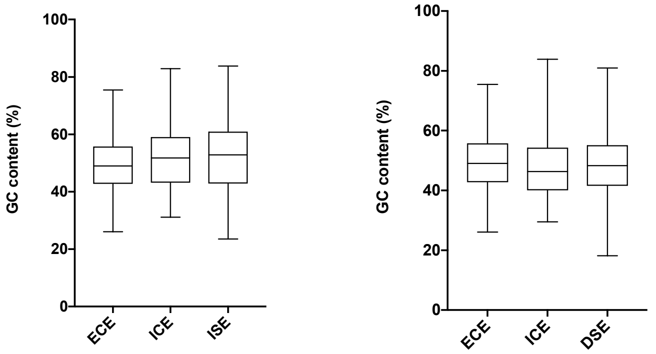

c)

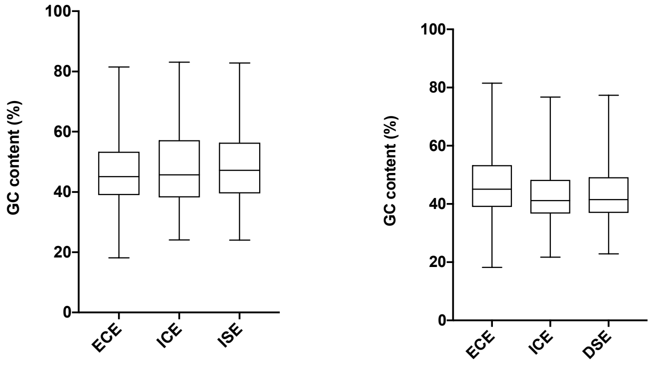

d)

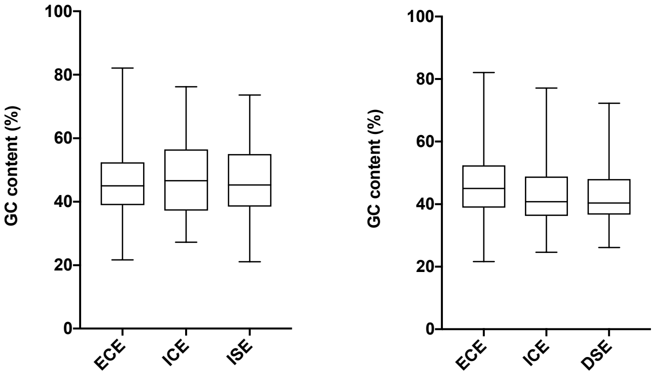

e)

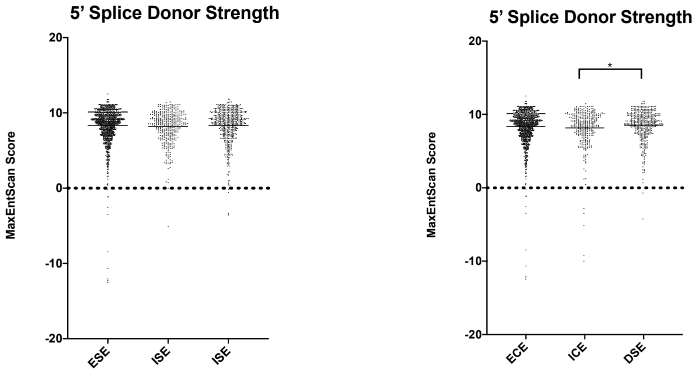

f)

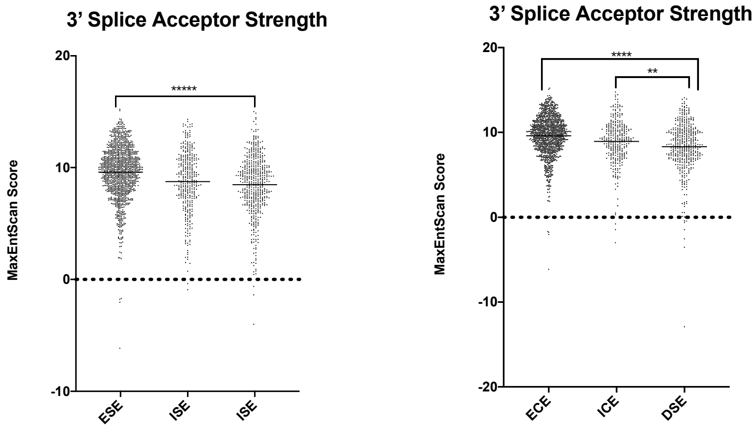

g)

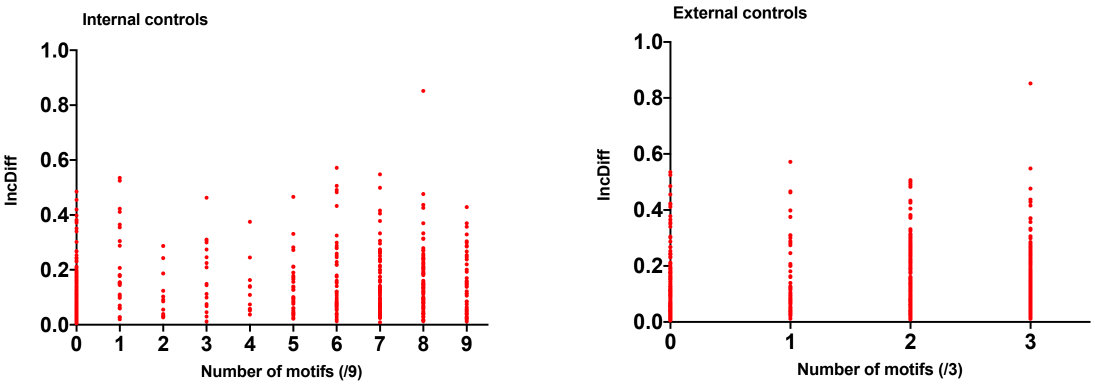

Supplementary Figure 8. Characteristics of skipped exons.
a) Length and **b)** GC content of exons showing increased skipping (ISE) and exons showing decreased skipping (DSE) in 3.C9 cells, relative to external control exons (ECE) and internal control exons (ICE). GC content of **c)** upstream and **d)** downstream introns surrounding exons showing increased skipping (ISE) and exons showing decreased skipping (DSE) in 3.C9 cells relative to external control exons (ECE) and internal control exons (ICE). Splice site strengths of **e)** upstream 5' splice donors and **f)** downstream 3' splice acceptors for exons showing

increased skipping (ISE) and exons showing decreased skipping (DSE) in 3.C9 cells, compared to external control exons (ECE) and internal control exons (ICE). **g)** Correlation between number of enriched motifs in skipped exon E-I-E-I-E sequences and severity of exon skipping for exons displaying increased skipping in 3.C9 cells. * p-value < 0.05. ** p-value < 0.01. **** p-value < 0.0001.

SUPPLEMENTARY TABLE 1

Number of Mis-Splicing Events	Genes	Types of mis-splicing
4	*GAS5*	RI, A5SS, MXE, SE
	RSRC2	RI, A3SS, MXE, SE
3	*EXOG*	A5SS, MXE, SE
	GCDH	RI, A5SS, MXE
	IDH3B	RI, A5SS, MXE
	MAN2C1	A3SS, MXE, SE
	PPOX	RI, MXE, SE
	RAB15	RI, A5SS, MXE
	SLC44A2	RI, A5SS, SE
	SNHG12	RI, A5SS, MXE
	SNHG5	A5SS, MXE, SE
	SRSF5	RI, A3SS, MXE
	TPM3	RI, MXE, SE
	TSC1	RI, MXE, SE
	WDR90	A5SS, A3SS, SE
2	*ABCB6*	RI, SE
	ADARB1	MXE, SE
	ADCK3	MXE, SE
	AKAP8L	A5SS, SE
	APOPT1	MXE, SE
	ARHGAP33	A3SS, SE
	ARHGEF9	MXE, SE
	ARL16	A5SS, SE
	BBS1	RI, A5SS
	BLVRB	MXE, SE
	BRD8	A5SS, MXE
	BTN3A3	RI, SE
	C14orf2	MXE, SE
	C17orf49	A5SS, SE
	C3orf17	RI, A5SS
	CADM1	MXE, SE
	CCDC189	RI, SE
	CHCHD7	RI, SE
	CLCN7	RI, A5SS
	CLK1	RI, SE
	CLK4	RI, A5SS
	CLMN	RI, A5SS
	CLSTN1	MXE, SE

	COL4A5	MXE, SE
	CTC-499B15.5	MXE, SE
	CTNND1	A5SS, SE
	CTPS1	RI, SE
	DDX17	RI, SE
	DDX55	RI, SE
	DEPDC5	A3SS, MXE
	DLST	MXE, SE
	DNAH14	MXE, SE
	DPF1	A5SS, SE
	ECT2	MXE, SE
	EHMT1	A3SS, SE
	EIF4A2	RI, SE
	ELAVL2	A3SS, SE
	EPB41L2	MXE, SE
	EPB41L3	A3SS, SE
	EVI5	MXE, SE
	EWSR1	RI, A5SS
	EXOC2	MXE, SE
	FAM49B	A5SS, SE
	FASTK	A5SS, SE
	FGFR3	MXE, SE
	FKTN	MXE, SE
	FOXM1	MXE, SE
	FUBP1	MXE, SE
	GOLT1B	MXE, SE
	GRK4	MXE, SE
	GTF3C1	RI, SE
	H2AFY	MXE, SE
	HACD3	MXE, SE
	HDAC10	MXE, SE
	IL17RC	A3SS, SE
	INO80E	A3SS, SE
	IQCH	MXE, SE
	JPX	MXE, SE
	KCNN1	MXE, SE
	KCTD19	RI, SE
	KDM2B	RI, SE
	KIF21A	A5SS, SE
	KIF23	MXE, SE
	LMBR1L	A3SS, SE

	LRRFIP2	MXE, SE
	LSM14A	MXE, SE
	MADD	MXE, SE
	MAPT	MXE, SE
	MELK	MXE, SE
	MFSD8	A3SS, SE
	MRPL48	MXE, SE
	MSH5	A3SS, SE
	MYL6	MXE, SE
	NDUFC1	MXE, SE
	NDUFS7	RI, A3SS
	OGFOD2	A5SS, SE
	PBRM1	MXE, SE
	PDDC1	A5SS, SE
	PGAP2	MXE, SE
	PHLDB1	RI, SE
	PLOD2	MXE, SE
	PPIEL	A5SS, A3SS
	RAD17	MXE, SE
	RBM3	A5SS, SE
	REPS1	A5SS, SE
	RGP1	A5SS, SE
	RNF34	MXE, SE
	RP1-34B21.6	A3SS, SE
	RP11-295P9.3	MXE, SE
	RP11-322M19.1	A3SS, SE
	RP11-383H13.1	MXE, SE
	RP11-46C20.1	A3SS, SE
	RP4-717I23.3	MXE, SE
	RPS24	MXE, SE
	SAMD11	MXE, SE
	SCML1	MXE, SE
	SERAC1	A3SS, SE
	SFXN2	MXE, SE
	SLC25A3	RI, A5SS
	SLC9A5	A5SS, SE
	SMARCC2	A5SS, SE
	SNAP91	MXE, SE
	SNHG8	A5SS, SE
	SPAG9	MXE, SE
	SRRM1	RI, SE

	SRSF7	RI, SE
	SS18L1	MXE, SE
	STARD4	A5SS, SE
	STAT2	RI, SE
	SYNE2	MXE, SE
	SYT14	MXE, SE
	TAF1D	RI, MXE
	TCIRG1	RI, SE
	TMBIM6	MXE, SE
	TMEM150A	RI, A5SS
	TONSL	A3SS, SE
	TPM1	MXE, SE
	TXNRD1	MXE, SE
	VPS13B	MXE, SE
	WARS	MXE, SE
	ZBED3-AS1	MXE, SE
	ZFAND6	MXE, SE
	ZFP90	A5SS, SE
	ZNF195	A5SS, SE
	ZNF248	RI, SE
	ZNF419	A3SS, SE
	ZSWIM7	RI, SE

Supplementary Table 1. Numbers and identities of genes displaying more than one class of altered splicing in 3.C9 cells obtained using rMATS v.4.0.2. RI = intron retention, A5SS = alternative 5’ splice site, A3SS = alternative 3’ splice site, MXE = mutually exclusive exons, SE = exon skipping.

SUPPLEMENTARY TABLE 2

Gene class	Top upstream regulators	Top associated networks
**All mis-spliced genes**	Monobutyl phthalate	Cell Death and Survival, Auditory Disease, Developmental Disorder
	GnRH analog	RNA Post-Transcriptional Modification, Molecular Transport, RNA Trafficking
	TP53	Developmental Disorder, Hereditary Disorder, Neurological Disease
	MAFF	Cell Cycle, Neurological Disease, Organ Morphology
	Methylselenic acid	Developmental Disorder, Hereditary Disorder, Metabolic Disease
	MEP1B	Cellular Assembly and Organization, Cellular Compromise, Cell-To-Cell Signaling and Interaction
	ADCY5	Cellular Assembly and Organization, Nervous System Development and Function, Organ Morphology
	APBA1	Cellular Compromise, Cell Cycle, Cellular Assembly and Organization
	PLPP3	Cellular Movement, Embryonic Development, Nervous System Development and Function
	GGA2	Gene Expression, Developmental Disorder, Hereditary Disorder
**All SE genes**	TP53	Auditory Disease, Developmental Disorder, Hereditary Disorder
	ADCY5	Cardiovascular Disease, Cardiovascular System Development and Function, Cell Morphology
	APBA1	RNA Post-Transcriptional Modification, Molecular Transport, RNA Trafficking
	GGA2	Post-Translational Modification, Developmental Disorder, Embryonic Development
	DRP2	Cell Cycle, Cellular Assembly and Organization, DNA Replication, Recombination, and Repair
	SLC6A2	Cell Morphology, Cellular Assembly and Organization, Developmental Disorder
	HGF	Hematological Disease, Immunological Disease, Cancer
	Monobutyl phthalate	Lipid Metabolism, Molecular Transport, Small Molecule Biochemistry
	Methylselenic acid	Cellular Assembly and Organization, Cancer, Endocrine System Disorders
	MEP1B	RNA Post-Transcriptional Modification, Cell Cycle, Cellular Development
	TP53	Cancer, Gastrointestinal Disease, Organismal Injury and Abnormalities

**Genes with increased SE in 3.C9**	ACACB	Protein Trafficking, Cardiovascular Disease, Connective Tissue Disorders
	ESRP1	Nucleic Acid Metabolism, Small Molecule Biochemistry, Hereditary Disorder
	GNE	Cell Cycle, Amino Acid Metabolism, Cellular Assembly and Organization
	HIST4H4	Gene Expression, RNA Post-Transcriptional Modification, Hereditary Disorder
	ACACA	Cellular Assembly and Organization, Cellular Function and Maintenance, Cellular Movement
	torin1	Lipid Metabolism, Small Molecule Biochemistry, Vitamin and Mineral Metabolism
	E2F5	Bradycardia, Cardiac Arrythmia, Cardiovascular Disease
	Methylselenic acid	Cell Cycle, Embryonic Development, Cellular Assembly and Organization
	MEP1B	Cellular Development, Cellular Growth and Proliferation, Connective Tissue Disorders
**Genes with decreased SE in 3.C9**	SLC6A2	Auditory Disease, Cell Morphology, Cellular Compromise
	Camptothecin	RNA Post-Transcriptional Modification, Molecular Transport, RNA Trafficking
	APBA1	Cellular Assembly and Organization, Cellular Function and Maintenance, Cancer
	DRP2	Developmental Disorder, Hereditary Disorder, Organismal Injury and Abnormalities
	SLC18A2	Cancer, Organismal Injury and Abnormalities, Cell Morphology
	SRSF2	Connective Tissue Development and Function, Connective Tissue Disorders, Nervous System Development and Function
	HTR6	Cellular Assembly and Organization, Cellular Function and Maintenance, Cardiovascular Disease
	SNRNP70	Cellular Assembly and Organization, Cellular Function and Maintenance, Tissue Development
	mir-210	Molecular Transport, Cell Morphology, Nervous System Development and Function
	Methylene blue	Gastrointestinal Disease, Hepatic System Disease, Organismal Injury and Abnormalities

Supplementary Table 2. Top 10 upstream regulators and top 10 functional networks associated with all significantly mis-spliced genes, all genes displaying altered exon skipping (SE) events, genes displaying significantly increased exon skipping events in 3.C9 cells and genes displaying significantly decreased exon skipping events in 3.C9 cells, obtained using Ingenuity Pathway Analysis (IPA).

SUPPLEMENTARY TABLE 3

RI group	Control set used	Motif	E-value	Number of sites
**Introns more retained in 3.C9**	Internal	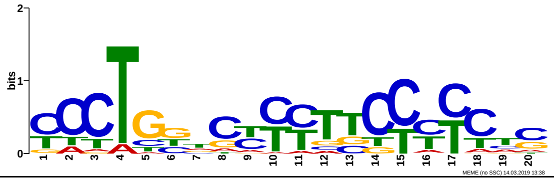	5.4E-22	84/84
		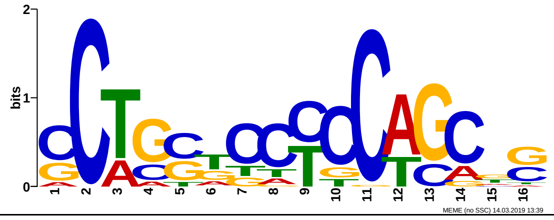	2.8E-13	57/84
		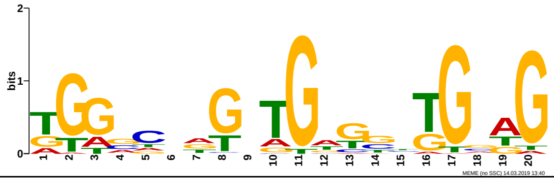	6.9E-06	84/84
	External	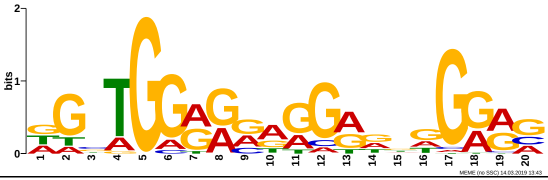	1.2E-30	72/84
		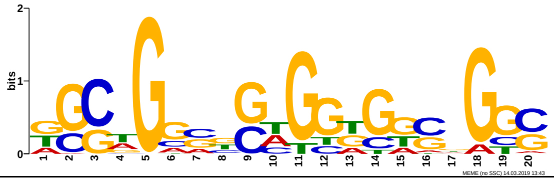	1.6E-06	53/84
		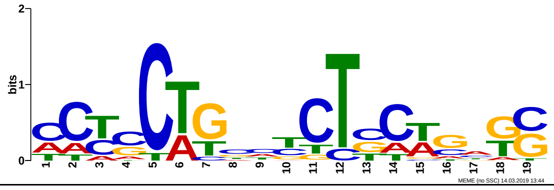	4.8E-04	84/84
		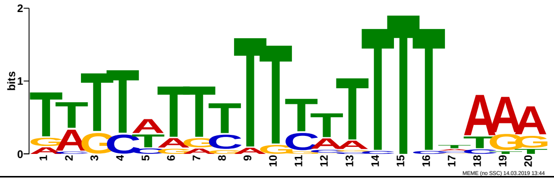	6.0E-09	33/84
**Introns less retained in 3.C9**	Internal	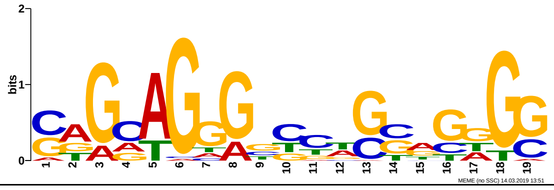	4.3E-11	50/50

		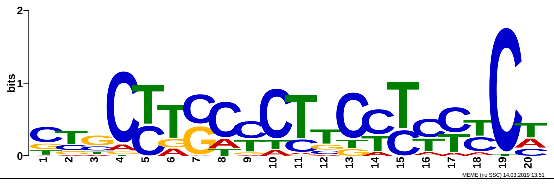	4.4E-18	50/50
		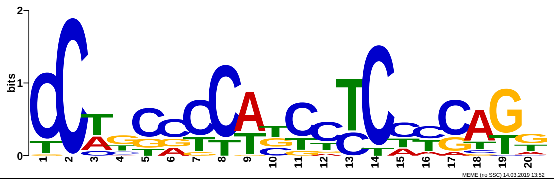	8.8E-03	38/50
		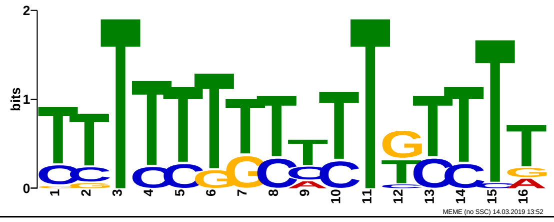	1.8E-07	24/50
	External	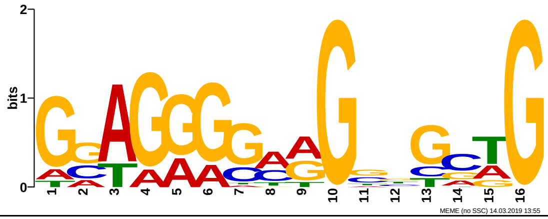	1.2E-16	50/50
		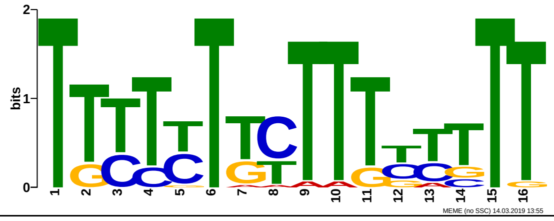	8.5E-12	21/50
		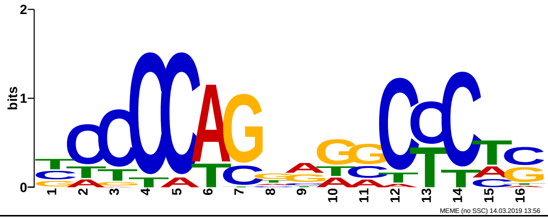	1.0E-07	50/50
		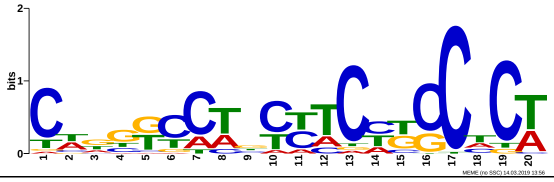	2.1E-05	50/50
		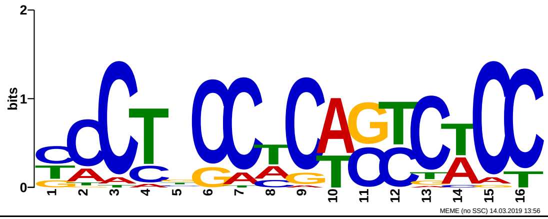	1.6E-02	35/50

Supplementary Table 3. Significant (E-value < 0.05) enriched 5 – 20 nucleotide sequence motifs in intron plus up- and downstream flanking exon (E-I-E) sequences for introns more

retained in 3.C9 cells and introns less retained in 3.C9 cells, using either extragenic or intragenic E-I-E controls as background sequences, and the statistical significance (E-value) of enriched motifs and number of occurrences in retained intron input sequences obtained using the discriminative mode of the MEME tool in the MEME suite package.

SUPPLEMENTARY TABLE 4

SE group	Control set used	Motif	E-value	Number of sites
**Exons more skipped in 3.C9**	Internal	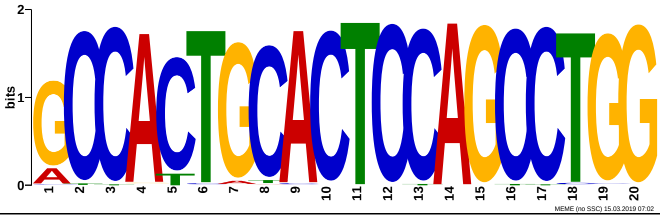	1.8E-10	314/567
		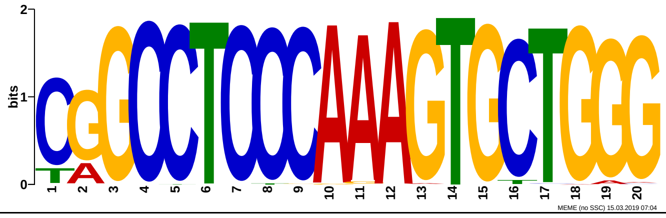	1.8E-10	347/567
		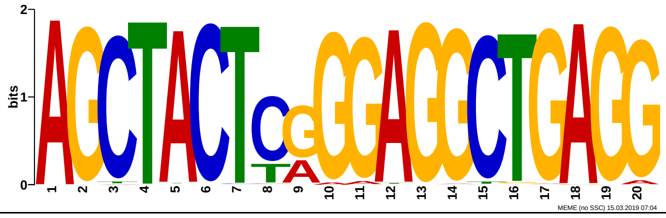	1.2E-08	331/567
		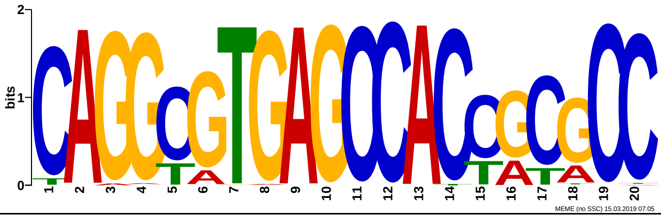	3.2E-08	320/567
		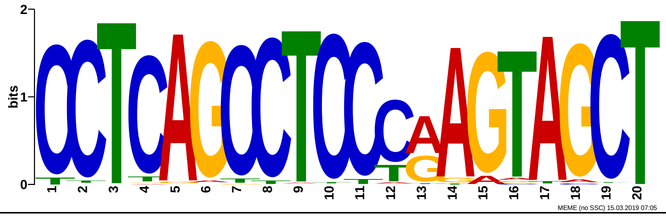	1.4E-05	264/567
		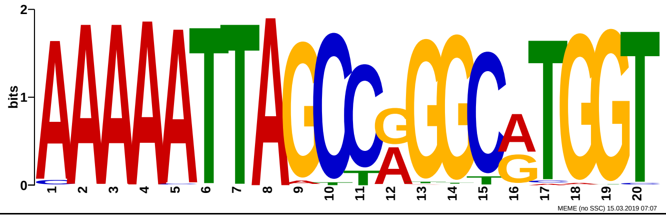	1.4E-04	234/567
		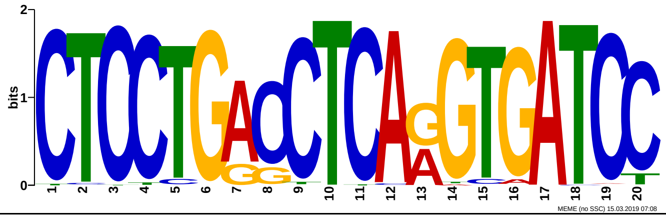	8.7E-07	304/567

		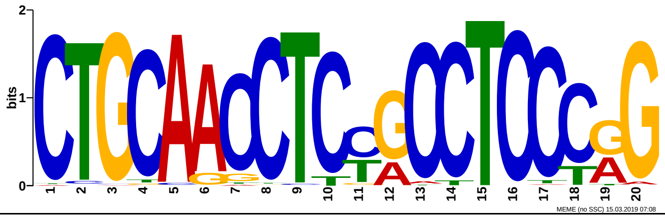	6.5E-04	350/567
		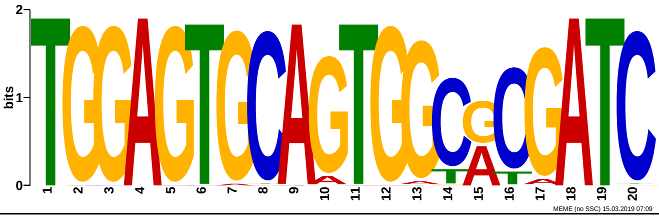	2.1E-02	113/567
	External	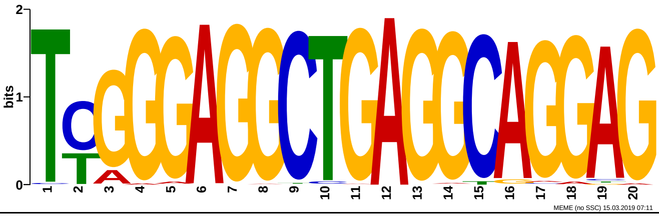	1.2E-08	344/567
		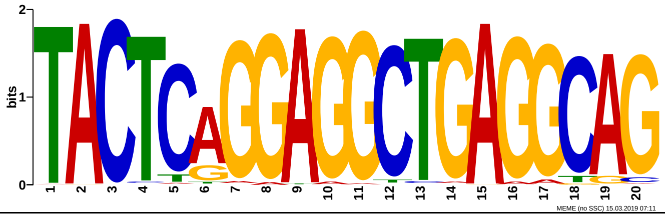	1.3E-07	249/567
		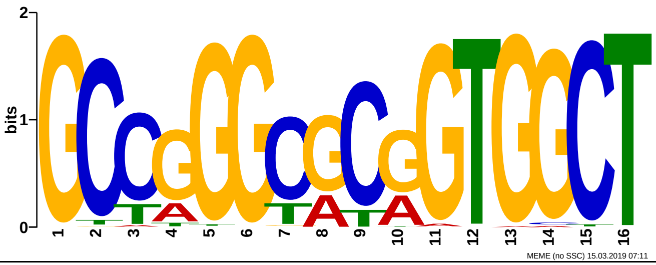	2.6E-02	285/567
**Exons less skipped in 3.C9**	Internal	No enriched motifs	N/A	N/A
	External	No enriched motifs	N/A	N/A

Supplementary Table 4. Significant (E value < 0.05) enriched 5 – 20 nucleotide sequence motifs in exon plus up- and downstream flanking intron and exon (E-I-E-I-E) sequences for exons more skipped in 3.C9 cells and exons less skipped in 3.C9 cells, using either extragenic or intragenic E-I-E-I-E controls as background sequences, and the statistical significance (E-value) of enriched motifs and number of occurrences in skipped exon input sequences obtained using the discriminative mode of the MEME tool in the MEME suite package.

SUPPLEMENTARY PRIMER TABLES

SUPPEMENTARY PRIMER TABLE 1: Oligonucleotides for gRNA Cloning for CRISPR-Cas9n System

Cas9 nickase target site	sgRNA oligo	Oligonucleotide sequence (5' – 3')
EFTUD2 exon 4	1A	CACCGGGAGCAGACATTACCTGTTA
	1B	AAACTAACAGGTAATGTCTGCTCCC
	2A	CACCGGTTTTCACTGGCTTAATAAT
	2B	AAACATTATTAAGCCAGTGAAAACC

SUPPLEMENTARY PRIMER TABLE 2: Primer sequences for PCR reactions to screen for successful CRISPR Mutants

Target	Primer	Primer sequence (5' – 3')	Wildtype product length
EFTUD2 exon 4	Forward	TTTATGCCCACCTACACATCAC	180bp
	Reverse	TACAAAAACATGAACCCACAGC	

SUPPLEMENTARY PRIMER TABLE 3: Primer sequences for cloning and sequencing of CRISPR mutants

Target	Primer	Primer sequence (5' – 3')
EFTUD2 exon 4	Forward	CGGAATTCTTTATGCCCACCTACACATCAC
	Reverse	GGGGTACCTACAAAAACATGAACCCACAGC

SUPPLEMENTARY PRIMER TABLE 4: Primers for qPCR and RT-PCRs

Target Gene	Forward primer sequence (5' – 3')	Reverse primer sequence (5' – 3')
ACTB	GTGGATCAGCAAGCAGGAGT	GTAACAACGCATCTCATATTTGGAA
EFTUD2	GACAGCAACCATAACCGAACC	ATGTCTATCTCTGAGTACATCTTCCG
UPF1	GGAGAAAACACCTAACATCACCA	CCTTGTCAGTGAAAATCCCAACT
CNB1	GGAGAAAACACCTAACATCACCA	CCTTGTCAGTGAAAATCCCAACT
ERN1	CACAGTGACGCTTCCTGAAAC	GCCATCATTAGGATCTGGGAGA
PERK	ACGATGAGACAGAGTTGCGAC	ATCCAAGGCAGCAATTCTCCC
ATF6	TCCTCGGTCAGTGGACTCTTA	CTTGGGCTGAATTGAAGGTTTTG
XBP1	TTACGAGAGAAAACTCATGGCC	GGGTCCAAGTTGTCCAGAATGC
ABCB6 E4-E6	CTTGCTGACTGAGAAGGCACC	CATGCACAGGAACACAATGAGG
ABCB6 E16 - 17	GGACTGAAGCTGAGCGGCG	TGGTGCGGTTGGCACAGAC
LSM14A E6-E8	GTACACAAAGTTTCAAGGCCAG	AATAGCAATTAGGTCCAAGTGG
MRC2 E7-E9	CTTCCAGGGCCACTGCTACC	GATGGCAAGGACTGGTTACAGG
SCN2A E4-E7	ATGCTCATTATGTGCACGATTC	ACAGTCAAGATCATGACATCAG
